# UBE2C-induced crosstalk between mono- and polyubiquitination of SNAT2 promotes lymphatic metastasis in bladder cancer

**DOI:** 10.1172/JCI179122

**Published:** 2024-07-01

**Authors:** Wenjie Li, Changhao Chen, Hanhao Zheng, Yan Lin, Mingjie An, Daiyin Liu, Yonghai Zhang, Mingchao Gao, Tianhang Lan, Wang He

**Affiliations:** 1 Department of Urology, Sun Yat-sen Memorial Hospital, Sun Yat-sen University, Guangzhou, Guangdong, China.; 2 Guangdong Provincial Key Laboratory of Malignant Tumor Epigenetics and Gene Regulation, Guangdong-Hong Kong Joint Laboratory for RNA Medicinem, Guangdong, China.; 3 Guangdong Provincial Clinical Research Center for Urological Diseases, Guangdong, China.; 4 Department of Urology, Shantou Central Hospital, Shantou, Guangdong, China.

**Keywords:** Cell biology, Oncology, Cancer, Oncogenes, Ubiquitin-proteosome system

## Abstract

Ubiquitination plays an essential role in protein stability, subcellular localization, and interactions. Crosstalk between different types of ubiquitination results in distinct biological outcomes for proteins. However, the role of ubiquitination-related crosstalk in lymph node (LN) metastasis and the key regulatory factors controlling this process have not been determined. Using high-throughput sequencing, we found that ubiquitin-conjugating enzyme E2 C (*UBE2C*) was overexpressed in bladder cancer (BCa) and was strongly associated with an unfavorable prognosis. Overexpression of *UBE2C* increased BCa lymphangiogenesis and promoted LN metastasis both in vitro and in vivo. Mechanistically, UBE2C mediated sodium-coupled neutral amino acid transporter 2 (SNAT2) monoubiquitination at lysine 59 to inhibit K63-linked polyubiquitination at lysine 33 of SNAT2. Crosstalk between monoubiquitination and K63-linked polyubiquitination increased SNAT2 membrane protein levels by suppressing epsin 1–mediated (EPN1-mediated) endocytosis. SNAT2 facilitated glutamine uptake and metabolism to promote VEGFC secretion, ultimately leading to lymphangiogenesis and LN metastasis in patients with BCa. Importantly, inhibition of *UBE2C* significantly attenuated BCa lymphangiogenesis in a patient-derived xenograft model. Our results reveal the mechanism by which UBE2C mediates crosstalk between the monoubiquitination and K63-linked polyubiquitination of SNAT2 to promote BCa metastasis and identify UBE2C as a promising target for treating LN-metastatic BCa.

## Abstract 2

UBE2C mediates crosstalk between the monoubiquitination and K63-linked polyubiquitination of SNAT2 to promote bladder cancer lymphatic metastasis.

## Graphical abstract

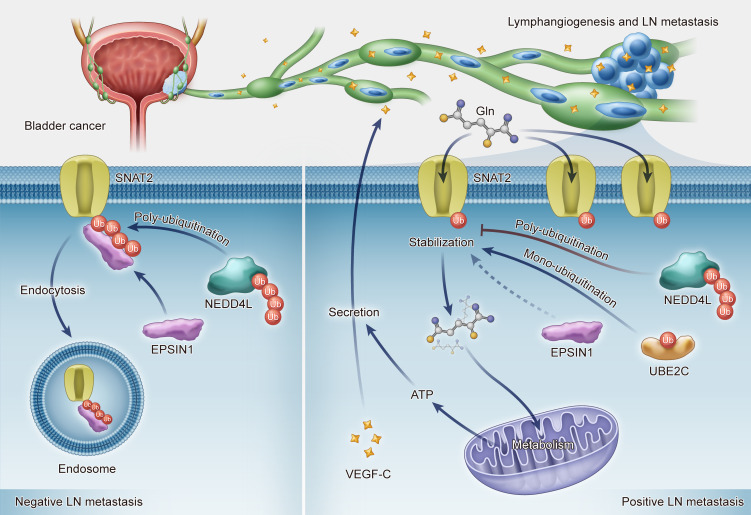

## Introduction

Lymph node (LN) metastasis is the predominant route of metastasis for most solid tumors and promotes distant metastasis (1, 2). Patients with LN involvement have shorter distant metastasis–free survival (DMFS) and overall survival (OS) than patients without LN involvement (3, 4). Extensive research has shown that lymphangiogenesis, a mechanism that generates new lymphatic vessels from preexisting lymphatic networks, plays a critical role in facilitating LN metastasis (5, 6). Newly formed lymphatic vessels not only serve as channels for tumor dissemination but also actively facilitate the recruitment of tumor cells into LNs, promoting the survival of tumor stem cells and regulating immune responses (7). Thus, a comprehensive investigation into the regulatory mechanisms of lymphangiogenesis has important clinical implications in terms of preventing tumor metastasis to LNs and subsequent distant spread.

Ubiquitination plays a crucial role in tumor metastasis by regulating protein degradation, subcellular localization, protein-protein interactions, and signal transduction (8–10). Given the way in which ubiquitin interacts with its substrates, ubiquitination modifications can be categorized into 3 distinct types: polyubiquitination, multiubiquitination, and monoubiquitination (11). The different types of ubiquitination result in distinct biological effects for substrate proteins: polyubiquitination primarily facilitates protein degradation and signal assembly (12, 13), whereas monoubiquitination predominantly modulates protein subcellular localization, interactions, and activity (14–16). Notably, the substrate ubiquitination type affects the ability of a protein to undergo another type of ubiquitination (17). Studies have shown that monoubiquitination of a substrate inhibits its polyubiquitination, increasing substrate stability and altering the substrate interaction network, leading to cancer progression (18, 19). However, the molecular mechanism underlying the crosstalk between monoubiquitination and polyubiquitination and its contribution to tumor LN metastasis are poorly understood.

Ubiquitination is catalyzed by a 3-enzyme cascade consisting of Ub-activating, Ub-conjugating (E2s), and Ub-ligating (E3s) enzymes (20). E3s have attracted substantial research attention due to their high substrate specificity, whereas E2s are often viewed simply as ubiquitin carriers that conjugate E3s to transfer ubiquitin to substrates (21). However, studies have shown that, in addition to acting as ubiquitin carriers, E2s can bind directly to target proteins and therefore play a role in determining where and how target proteins are modified by ubiquitin (22, 23). Therefore, aberrantly elevated expression of E2s in tumor cells may alter the pattern of substrate ubiquitination (24, 25). However, whether E2s mediate the crosstalk between different ubiquitination types to promote tumor LN metastasis remains to be determined.

In this study, we demonstrated that ubiquitin-conjugating enzyme E2 C (*UBE2C*) was upregulated in bladder cancer (BCa) and was positively associated with lymphangiogenesis and LN metastasis. UBE2C mediated sodium-coupled neutral amino acid transporter 2 (SNAT2) monoubiquitination at lysine 59 to inhibit K63-linked polyubiquitination at lysine 33 of SNAT2 by blocking the interaction between SNAT2 and NEDD4-like E3 ubiquitin protein ligase (NEDD4L). Crosstalk between monoubiquitination and K63-linked polyubiquitination increased the membrane expression level of SNAT2 by inhibiting epsin 1–mediated (EPN1-mediated) endocytosis and subsequently promoted glutamine uptake and metabolism. Moreover, increased glutamine metabolism facilitated VEGFC secretion, which in turn promoted BCa lymphangiogenesis and LN metastasis. This study reveals a mechanism of UBE2C in the induction of crosstalk between the monoubiquitination and K63-linked polyubiquitination of SNAT2, suggesting the potential of UBE2C as a promising therapeutic target for the treatment of LN-metastatic BCa.

## Results

### UBE2C is overexpressed in BCa and is positively associated with LN metastasis and lymphangiogenesis.

**Figure 1:**
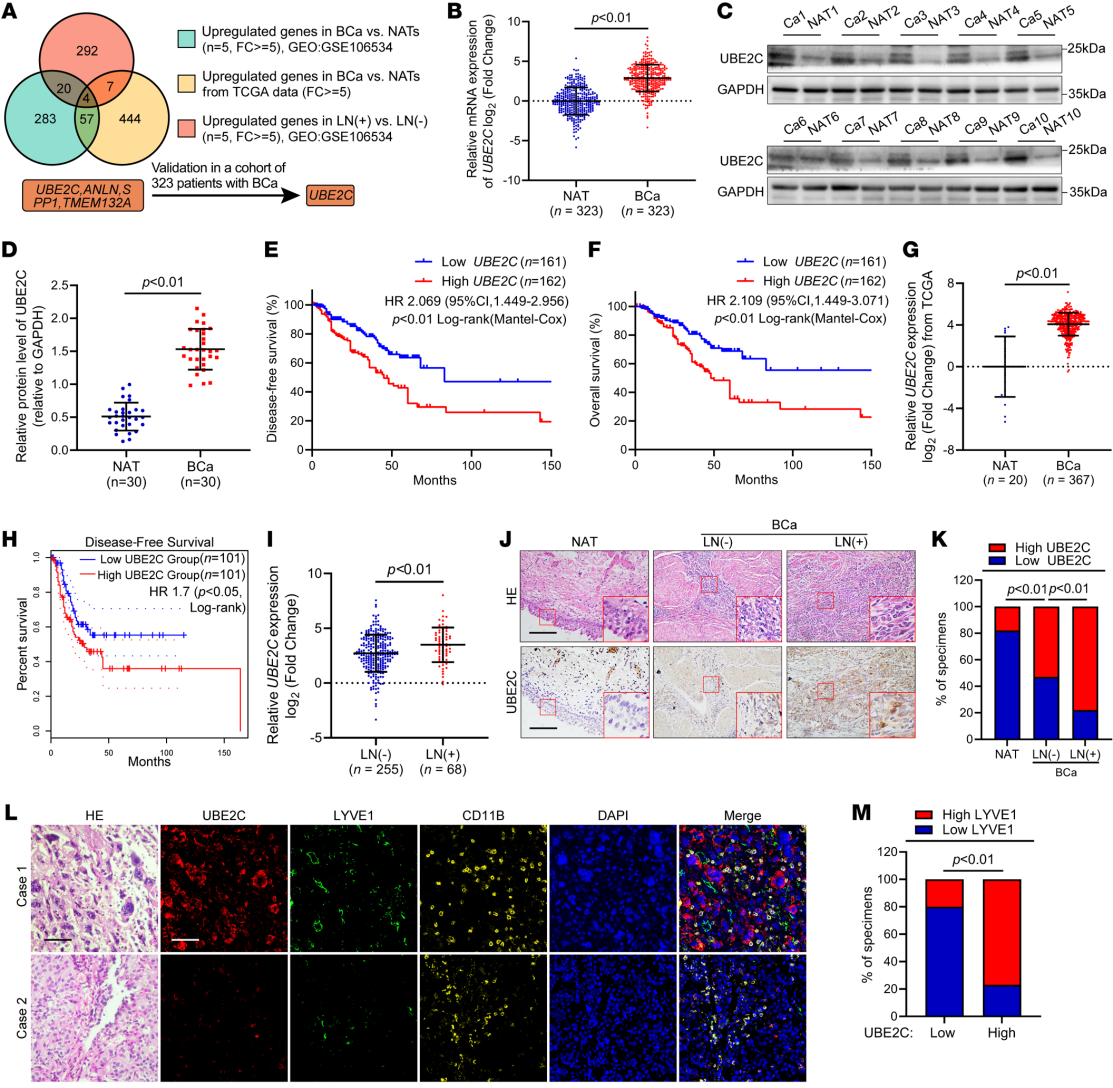
UBE2C expression is positively correlated with LN metastasis in patients with BCa. (**A**) Schematic representation of the process of screening genes upregulated in both BCa tissues and LN-positive BCa tissues. (**B**) qRT-PCR analysis of UBE2C expression in BCa tissues versus NATs (*n* = 323). (**C**) Representative immunoblot (IB) images of UBE2C expression in BCa tissues versus NATs. (**D**) Quantification of UBE2C expression in 30 paired BCa tissues and NATs by IB analysis. (**E** and **F**) K-M survival analysis of DFS (**E**) and OS (**F**) of patients with BCa with low versus high UBE2C expression. The cutoff was the median. (**G**) Analysis of UBE2C expression in BCa tissues and NATs retrieved from TCGA database. (**H**) K-M survival analysis of the DFS of BCa patients with low versus high UBE2C expression from TCGA database. The cutoff was the quartile. (**I**) qRT-PCR analysis of UBE2C expression in LN-positive versus LN-negative BCa (*n* = 323). (**J** and **K**) Representative IHC images and quantification of UBE2C expression in NATs and LN-negative and LN-positive BCa tissues. Scale bars: 50 μm. Original magnification, ×3 (enlarged insets). (**L** and **M**) Representative IF images and quantification of UBE2C expression and MLD indicated by LYVE1 and myeloid cells indicated by CD11B staining in BCa tissues. Scale bars: 50 μm. Significant differences were identified through the nonparametric Mann-Whitney *U* test (**B**, **D**, **G**, and **I**) and the χ^2^ test (**K** and **M**). Quantitative results are presented as the mean ± SEM of 3 separate experiments.

To investigate the critical genes contributing to BCa metastasis, we conducted next-generation sequencing (NGS) of 5 paired BCa tissues and their corresponding normal adjacent tissues (NATs) (Gene Expression Omnibus [GEO] GSE106534). In total, 364 genes were found to be upregulated more than 5-fold in BCa tissues compared with NATs [fold change (FC) >5, *P* < 0.01]. We also performed NGS of 5 LN-negative and 5 LN-positive BCa tissues (GEO GSE106534) and found that the expression of 323 genes was upregulated more than 5-fold in the LN-positive BCa tissues compared with the LN-negative tissues (FC >5, *P* < 0.01). In addition, analysis of The Cancer Genome Atlas (TCGA) database revealed that, compared with expression in normal tissues, the expression of 512 genes was upregulated more than 5-fold in BCa tissues (FC >5, *P* < 0.01). Next, we intersected these 3 sequencing data sets and identified 4 genes, including *UBE2C, ANLN, SPP1*, and *TMEM132A,* that were consistently upregulated in BCa tissues compared with expression in NATs and in BCa tissues from patients with LN metastasis compared with those without LN metastasis (Figure 1A). Subsequently, expression of the above 4 genes was validated in our large cohort composed of 323 patients with BCa, revealing significant overexpression of *UBE2C* in BCa tissues compared with expression in NATs (Figure 1B and Supplemental Figure 1, A–C; supplemental material available online with this article; https://doi.org/10.1172/JCI179122DS1). Moreover, higher protein levels of UBE2C were detected in BCa tissues than in NATs (Figure 1, C and D). *UBE2C* was overexpressed in both high-grade and high-T-stage BCa tissues compared with the control tissue (Supplemental Figure 1, D and E). K-M analysis demonstrated a significant positive correlation between *UBE2C* overexpression and reduced disease-free survival (DFS) and OS in patients with BCa (Figure 1, E and F, and Supplemental Figure 1, F–K). Furthermore, both univariate and multivariate Cox analyses confirmed that *UBE2C* was an independent prognostic factor for DFS and OS in patients with BCa (Supplemental Tables 1–3). According to an analysis of TCGA database, *UBE2C* was upregulated in various cancers, including BCa and cervical squamous cell carcinoma, and was closely linked to a poor prognosis for patients (Figure 1, G and H, and Supplemental Figure 1, L–O).

In our large cohort, statistical analysis confirmed that the expression of *UBE2C* was higher in BCa tissues from patients with LN metastasis than in those from patients without LN metastasis (Figure 1I and Supplemental Figure 1, P–R). Furthermore, IHC analysis revealed that UBE2C was upregulated in BCa tissues from patients with LN metastasis compared with those from patients without LN metastasis (Figure 1, J and K). Immunofluorescence (IF) staining was subsequently performed in BCa tissues to identify the association between UBE2C overexpression and increased microlymphatic vessel density (MLD) indicated by the expression of lymphatic vessel endothelial hyaluronan receptor 1 (LYVE1), suggesting that UBE2C is associated with BCa lymphangiogenesis (Figure 1, L and M). Overall, these findings demonstrate that the expression of UBE2C is associated with LN metastasis in patients with BCa.

### UBE2C facilitates BCa lymphangiogenesis in vitro.

**Figure 2:**
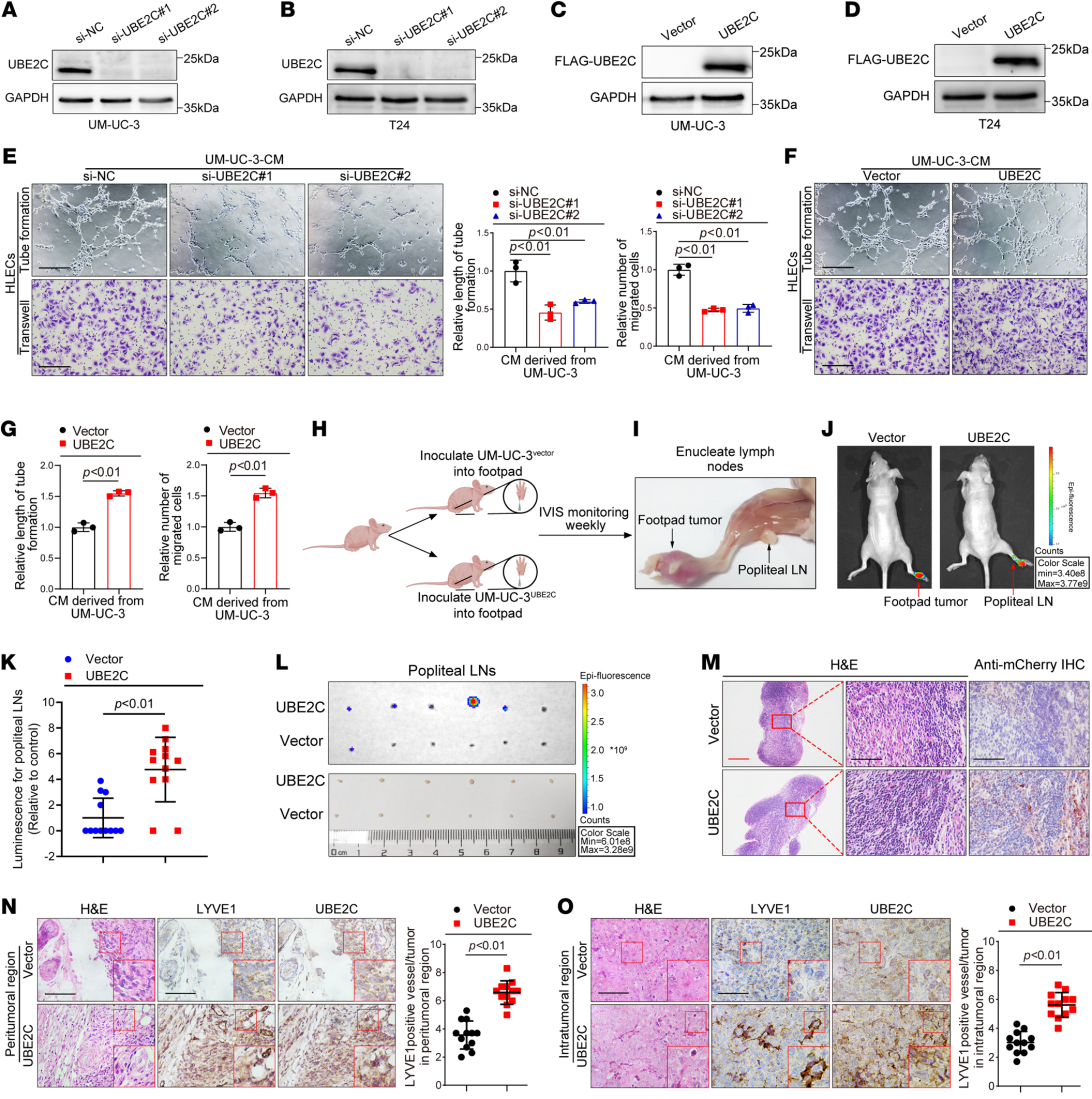
UBE2C promotes lymphangiogenesis and LN metastasis in BCa. (**A**–**D**) IB analysis of UBE2C expression following UBE2C overexpression or knockdown in BCa cells. (**E**–**G**) Representative images and quantification of tube formation and migration of HLECs after coculturing with UBE2C-knockdown or UBE2C-overexpressing UM-UC-3 cells. Scale bars: 100 μm. (**H** and **I**) Diagrammatic representation of the popliteal LN metastasis model using nude mice. (**J** and **K**) Representative images and quantification of bioluminescence in popliteal metastatic LNs (*n* = 12). Red arrows indicate footpad tumors and metastatic popliteal LNs. (**L**) Representative images and bioluminescence results for popliteal LNs from 2 groups of mice (*n* = 12). (**M**) Representative IHC images of anti–mCherry antibody–treated popliteal LNs from mice (*n* = 12). Red scale bar: 500 μm; black scale bars: 50 μm. (**N** and **O**) Representative IHC images and quantification of UBE2C expression and LYVE1-indicated MLD in the peritumoral (**N**) and intratumoral (**O**) regions of footpad tumor tissues. Scale bars: 50 μm. Significant differences were identified through 1-way ANOVA followed by Dunnett’s test (**E**) and 2-tailed Student’s *t* test (**G**, **K**, **N**, and **O**). Quantitative results are presented as the mean ± SEM of 3 separate experiments.

We investigated the biological function of UBE2C in regulating BCa lymphangiogenesis in vitro, which is a crucial step in tumor LN metastasis (26). First, we examined the expression of UBE2C in various BCa cell lines, and all of the cell lines had higher expression levels than in the human normal bladder epithelial cell lines (SV-HUC-1) (Supplemental Figure 2, A and B). Among the BCa cell lines, T24 and UM-UC-3 were selected because of their highly invasive and metastatic behavior; these cell lines were used to investigate the effect of UBE2C on lymphangiogenesis in vitro. Western blotting and quantitative reverse transcription PCR (qRT-PCR) analyses were performed to assess the transfection efficiency of the siRNAs targeting UBE2C and the UBE2C overexpression plasmid (Figure 2, A–D, and Supplemental Figure 2, C and D). Subsequently, human lymphatic endothelial cells (HLECs) were cocultured with UBE2C-overexpressing or UBE2C-knockdown T24 and UM-UC-3 cells to assess the effect of UBE2C on BCa lymphangiogenesis. We noted that tube formation and migration of HLECs were significantly suppressed after coculturing with UBE2C-knockdown UM-UC-3 and T24 cells (Figure 2E and Supplemental Figure 2E). In contrast, coculturing of HLECs with UBE2C-overexpressing UM-UC-3 and T24 cells strongly promoted the tube formation and migration of HLECs (Figure 2, F and G, and Supplemental Figure 2F), suggesting that UBE2C overexpression promoted BCa lymphangiogenesis in vitro.

In addition to tumor cell–induced lymphangiogenesis, the invasive potential of tumor cells contributed to the development of tumor LN metastasis. Transwell and wound-healing assays revealed that UBE2C knockdown significantly suppressed invasion and migration in UM-UC-3 and T24 cells, whereas overexpression of UBE2C markedly promoted BCa cell invasion and migration (Supplemental Figure 2, G–L). In summary, the above results demonstrate that overexpression of UBE2C facilitated BCa lymphangiogenesis in vitro.

### UBE2C promotes BCa lymphangiogenesis and LN metastasis in vivo.

We established a model of footpad popliteal LN metastasis by inoculating mCherry-labeled UM-UC-3 cells with or without overexpression of UBE2C into the footpad of mice (Figure 2, H and I), as previously described (6), to investigate the effect of UBE2C on LN metastasis in BCa in vivo. Analysis with an in vivo imaging system (IVIS) revealed that, compared with the control treatment, UBE2C overexpression significantly increased the fluorescence intensity in popliteal LNs (Figure 2, J and K), suggesting that UBE2C facilitated BCa cell metastasis from the footpad to the popliteal LNs. Furthermore, IVIS and IHC analyses revealed that the popliteal LN metastasis rate was greater in the UBE2C-overexpressing group than in the control group (Figure 2, L and M, and Supplemental Table 4). Notably, UBE2C overexpression increased the peritumoral and intratumoral MLD compared with that in the control group (Figure 2, N and O, and Supplemental Figure 2, M and N), suggesting that UBE2C induced lymphangiogenesis in BCa in vivo. Taken together, these results confirm that UBE2C promoted BCa lymphangiogenesis and LN metastasis in vivo.

### UBE2C induces crosstalk between SNAT2 monoubiquitination and K63-linked polyubiquitination.

**Figure 3:**
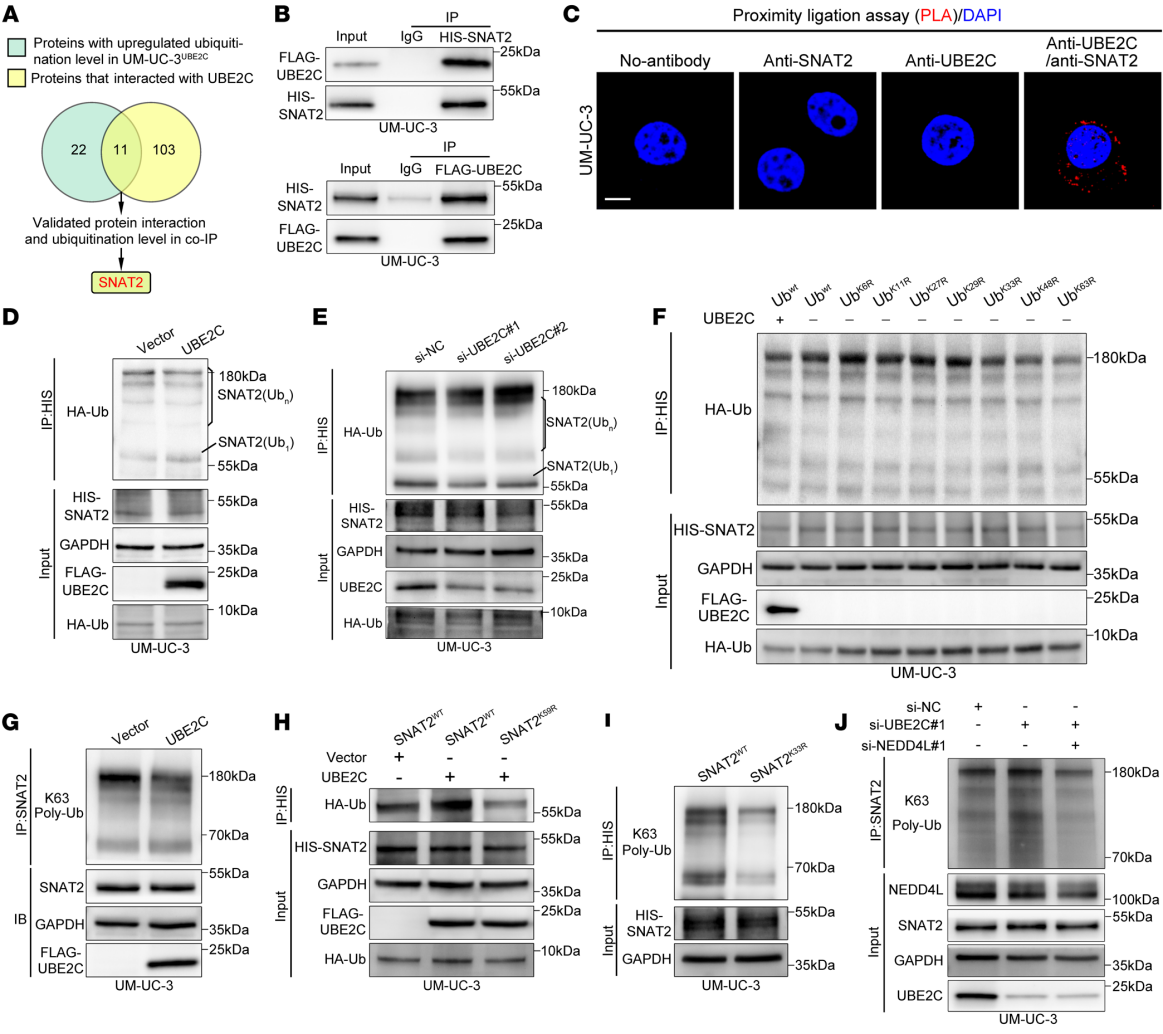
UBE2C promotes the monoubiquitination of SNAT2 to inhibit its K63-linked polyubiquitination. (**A**) Schematic representation of the screening process for ubiquitination substrates of UBE2C. (**B**) IB analysis after co-IP assays with anti-FLAG or anti-HIS in UM-UC-3 cells. (**C**) PLAs showing the interaction between UBE2C and SNAT2. Scale bars: 5 μm. (**D** and **E**) IB analysis validating the UBE2C-mediated ubiquitination of SNAT2. HA-Ub, hemagglutinin-ubiquitin. (**F**) IB analysis of polyubiquitination types inhibited by UBE2C. si-NC, small interfering normal control RNA. (**G**) IB analysis confirming that K63-linked polyubiquitination of SNAT2 was inhibited by UBE2C. (**H**) IB analysis revealing the monoubiquitination site on SNAT2. (**I**) IB analysis revealing the polyubiquitination site on SNAT2. (**J**) IB analysis of the K63-linked polyubiquitination level of SNAT2 after si-UBE2C and si-NEDD4L transfection.

To investigate the underlying mechanisms by which UBE2C overexpression promotes LN metastasis in BCa, we performed mass spectrometry (MS) analysis, which revealed that, compared with the control group, the overexpression of UBE2C increased the ubiquitination levels of 33 proteins. These upregulated proteins intersected with the UBE2C-interacting proteins identified by IP-MS to reveal substrates of UBE2C (Figure 3A). Among the proteins identified from the above screening, SNAT2 exhibited the most substantial increase in ubiquitination in the UBE2C-overexpressing group (Supplemental Figure 3). IF staining was performed to determine the intracellular locations of UBE2C and SNAT2. The results revealed that UBE2C was present in both the nucleus and cytoplasm, where it colocalized with SNAT2 (Supplemental Figure 4, A and B). Next, co-IP and proximity ligation assays (PLAs) confirmed the interaction between UBE2C and SNAT2 (Figure 3, B and C, and Supplemental Figure 4C). Western blot analysis revealed that overexpression of UBE2C promoted the monoubiquitination of SNAT2, whereas downregulation of UBE2C had the opposite effect (Figure 3, D and E, and Supplemental Figure 4D). Unexpectedly, we found that UBE2C promoted the monoubiquitination of SNAT2 but blocked ubiquitin chain extension (Figure 3, D and E, and Supplemental Figure 4D), indicating that UBE2C induced crosstalk between the monoubiquitination and polyubiquitination of SNAT2.

To further investigate the crosstalk between the ubiquitination types of SNAT2, we transfected ubiquitin mutants (with mutations to arginine [R] at different lysine [K] sites) to examine the polyubiquitination types of SNAT2. The results showed that the K63R mutation of ubiquitin significantly reduced the polyubiquitination level of SNAT2 (Figure 3F and Supplemental Figure 4E), indicating that SNAT2 polyubiquitination was K63 linked. Furthermore, Western blot analysis with a K63 linkage–specific polyubiquitin antibody consistently demonstrated that the inhibition of SNAT2 polyubiquitination by UBE2C was K63 linked (Figure 3G and Supplemental Figure 4F). Taken together, these findings suggest that UBE2C promoted the monoubiquitination of SNAT2 and blocked the extension of the K63-linked ubiquitin chain.

### UBE2C mediates monoubiquitination at lysine 59 to inhibit K63-linked polyubiquitination at lysine 33 of SNAT2.

We next investigated the specific lysine residue of SNAT2 that is targeted by UBE2C for ubiquitination. Ubiquitination profiling via MS elucidated an elevation in ubiquitination at lysine 59 and a reduction at lysine 33 on SNAT2 in UBE2C-overexpressed UM-UC-3 cells relative to the control group (Supplemental Figure 5, A–C). We replaced the lysine that might be modified by ubiquitin with arginine (SNAT2^K59R^ and SNAT2^K33R^) and subsequently subjected the proteins to co-IP. As shown in Figure 3H, SNAT2^K59R^ markedly suppressed UBE2C-mediated SNAT2 monoubiquitination, suggesting that SNAT2 was monoubiquitinated at the K59 residue. The SNAT2^K33R^ mutation blocked K63-linked polyubiquitination, suggesting that SNAT2 was polyubiquitinated at the K33 residue (Figure 3I). Accordingly, these results indicate that UBE2C facilitated the monoubiquitination of SNAT2 at residue K59 while inhibiting K63-linked polyubiquitination at residue K33.

A previous study reported that NEDD4-like E3 ubiquitin protein ligase (NEDD4L) promoted K63-linked polyubiquitination of SNAT2 (27). Therefore, we speculated that UBE2C inhibits K63-linked SNAT2 polyubiquitination by modulating the interaction between SNAT2 and NEDD4L. Our data confirmed the interaction of NEDD4L with SNAT2 (Supplemental Figure 5, D and E). Furthermore, the knockdown of NEDD4L eliminated the capacity of UBE2C silencing to promote the K63-linked polyubiquitination of SNAT2, while it did not affect the monoubiquitination of SNAT2 (Figure 3J and Supplemental Figure 5F). We also showed that overexpression of UBE2C blocked the interaction between SNAT2 and NEDD4L without affecting the expression of NEDD4L (Supplemental Figure 5G). After transfection of the SNAT2^K59R^-mutant plasmid into UM-UC-3 cells, co-IP revealed a substantial attenuation of the interaction between SNAT2 and NEDD4L (Supplemental Figure 5H), indicating that the K59 site was crucial for the interaction between SNAT2 and NEDD4L. Taken together, these results suggest that UBE2C promoted SNAT2 monoubiquitination at the K59 residue to prevent K63-linked polyubiquitination at K33 by blocking the interaction between SNAT2 and NEDD4L.

### Crosstalk between SNAT2 forms with monoubiquitination, and K63-linked polyubiquitination increases the SNAT2 membrane expression level by inhibiting endocytosis.

**Figure 4:**
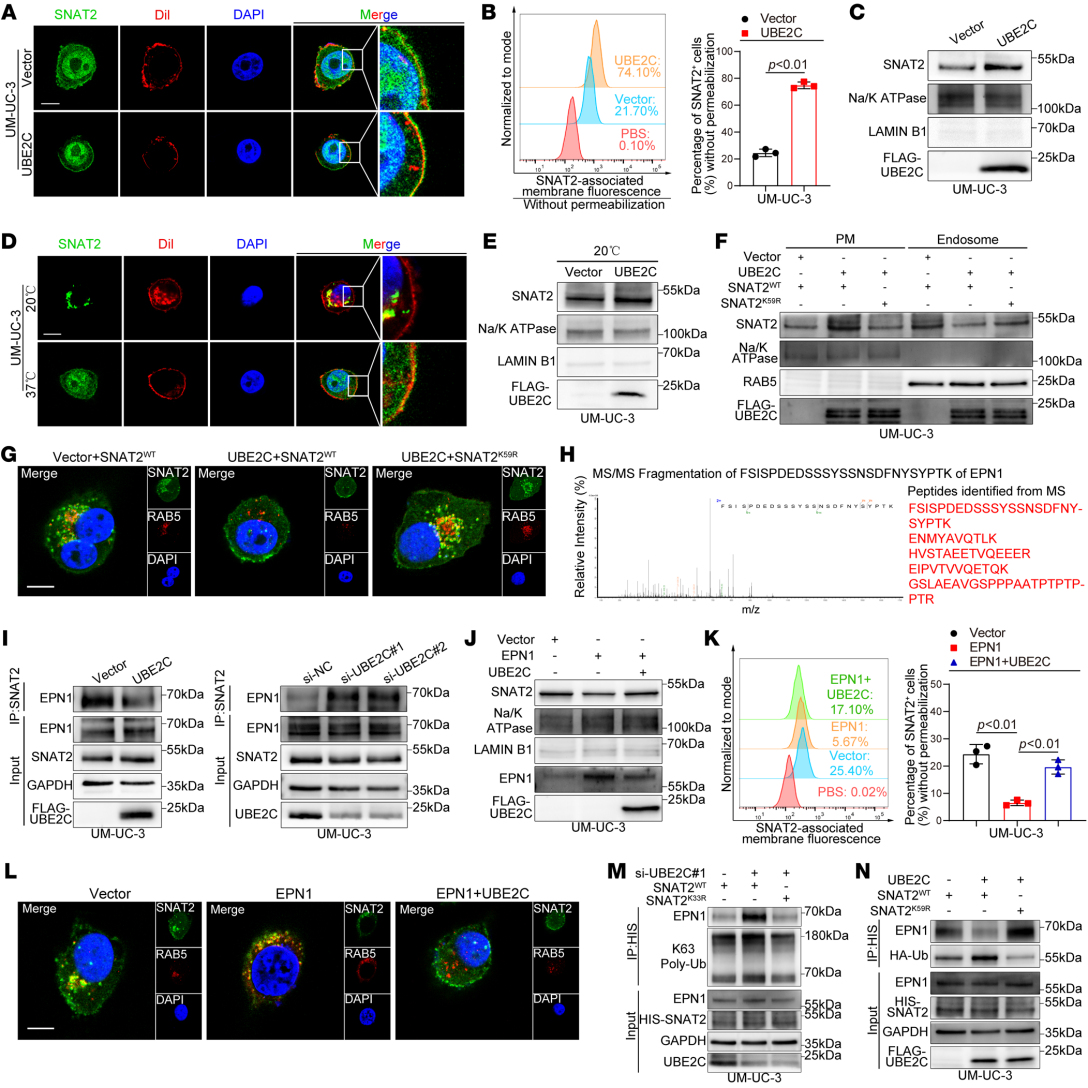
UBE2C increases the membrane expression level of SNAT2 by inhibiting its endocytosis. (**A**) IF assays showing the localization of SNAT2 after overexpression of UBE2C. Scale bar: 5 μm. Original magnification, ×4 (enlarged insets). (**B**) FACS analysis and quantification of SNAT2 expression in the membrane after overexpression of UBE2C. (**C**) IB analysis of SNAT2 expression in membrane fractions after overexpression of UBE2C. (**D**) IF analysis of the localization of SNAT2 in BCa cells subjected to cold blockade. Scale bar: 5 μm. Original magnification, ×4 (enlarged insets). (**E**) IB analysis of SNAT2 expression in membrane fractions after overexpression of UBE2C in cold blocks. (**F**) IB analysis of SNAT2 expression in membrane and endosome fractions after overexpression of UBE2C and SNAT2^K59R^ mutation. (**G**) IF assays showing the colocalization of SNAT2 and RAS oncogene family member (RAB5) after overexpression of UBE2C and the SNAT2^K59R^ mutation. Scale bar: 5 μm. (**H**) MS analysis for the detection of SNAT2-interacting proteins. (**I**) IB analysis of the interaction between SNAT2 and EPN1 after overexpression or knockdown of UBE2C. (**J** and **K**) IB (**J**) and FACS (**K**) analysis of SNAT2 expression in membrane fractions after overexpression of EPN1 and UBE2C. (**L**) IF assays showing the colocalization of SNAT2 and RAB5 after the overexpression of EPN1 and UBE2C. Scale bar: 5 μm. (**M**) IB analysis of the interaction between SNAT2 and EPN1 after SNAT2^K33R^ mutation. (**N**) IB analysis of the interaction between SNAT2 and EPN1 after SNAT2^K59R^ mutation. Significant differences were identified through 2-tailed Student’s *t* test (**B**) and 1-way ANOVA followed by Dunnett’s test (**K**). Quantitative results are presented as the mean ± SEM of 3 separate experiments.

Since ubiquitination normally acts as a signal for membrane protein degradation (28–30), we next investigated whether UBE2C promotes SNAT2 degradation. Unexpectedly, we found that neither overexpression nor knockdown of UBE2C affected SNAT2 protein levels (Supplemental Figure 6, A and B). Furthermore, the half-life of SNAT2 in UM-UC-3 cells remained consistent regardless of the UBE2C overexpression status (Supplemental Figure 6C), suggesting that UBE2C-induced monoubiquitination had no effect on SNAT2 degradation. Notably, IF staining indicated that SNAT2 was enriched on the cell membrane in the UBE2C-overexpressing group (Figure 4A). FACS analysis without permeabilization and Western blot analysis of the membrane fractions confirmed that overexpression of UBE2C increased the membrane expression level of SNAT2 (Figure 4, B and C, and Supplemental Figure 6, D and E), whereas inhibiting the monoubiquitination of SNAT2 by mutating K59 on SNAT2 eliminated these effects (Supplemental Figure 6, F–I), indicating that UBE2C-mediated monoubiquitination at the K59 residue increased the membrane expression level of SNAT2.

The expression level of membrane proteins on the cell surface are regulated by the dynamic equilibrium among exocytosis, recycling, and endocytosis (31). Consistent with previous research (32, 33), we observed that culturing at 20°C allowed endocytosis to continue but prevented protein exocytosis from the Golgi (Figure 4D). Next, FACS analysis without permeabilization and Western blot analysis of the membrane fractions revealed that overexpression of UBE2C still increased the membrane expression level of SNAT2 at 20°C (Figure 4E and Supplemental Figure 6J), suggesting that increased membrane SNAT2 expression in the UBE2C-overexpressing group was caused by the inhibition of endocytosis. Western blot analysis of the membrane and endosome fractions showed that expression of SNAT2 in the endosome fraction was decreased in UBE2C-overexpressing UM-UC-3 cells compared with control cells (Figure 4F). Furthermore, IF confirmed that overexpression of UBE2C inhibited the colocalization of SNAT2 with RAB5 (an early endosome marker) (Figure 4G), indicating that the increased expression of membrane SNAT2 in the UBE2C-overexpressing group was attributed to the inhibition of endocytosis.

To further elucidate the mechanisms by which UBE2C overexpression inhibits SNAT2 endocytosis, we performed co-IP and silver staining analyses to identify proteins that interact with SNAT2. Silver staining revealed that, compared with those in the control group, the proteins in the UBE2C-overexpressing group exhibited an obvious weak band with a molecular mass of 55–70 kDa (Supplemental Figure 6K), which was identified as EPN1 by MS (Figure 4H and Supplemental Figure 6L). Western blotting analysis following co-IP confirmed that the interaction between SNAT2 and EPN1 was attenuated in the UBE2C-overexpressing group but increased in the UBE2C-knockdown group (Figure 4I). EPN1 is an endocytic adapter protein that can bind ubiquitinated cargo to the clathrin-mediated endocytic machinery to promote endocytosis (34). Therefore, we postulated that UBE2C overexpression inhibits SNAT2 endocytosis by affecting the interaction between SNAT2 and EPN1. Overexpression of EPN1 strongly facilitated the endocytosis of SNAT2, whereas overexpression of UBE2C abrogated this effect (Figure 4, J–L, and Supplemental Figure 6, M and N), suggesting that overexpression of UBE2C prevented SNAT2 endocytosis by blocking the interaction between SNAT2 and EPN1. EPN1 has been reported to favor binding to polyubiquitin, which has a stronger endocytic signal than monoubiquitin (35). Therefore, we separately transfected SNAT2 plasmids with K33 or K59 mutated to arginine, which were previously shown to be sites of poly- or monoubiquitination. Our data confirmed that the altered interaction between SNAT2 and EPN1 was triggered by UBE2C-induced crosstalk between the monoubiquitinated and K63-linked polyubiquitinated forms of SNAT2 (Figure 4, M and N). Taken together, these findings indicate that UBE2C-mediated crosstalk between the monoubiquitinated and polyubiquitinated forms upregulated the membrane expression level of SNAT2 through the inhibition of endocytosis.

### Membrane-enriched SNAT2 promotes the secretion of VEGFC by increasing glutamine uptake and metabolism.

**Figure 5:**
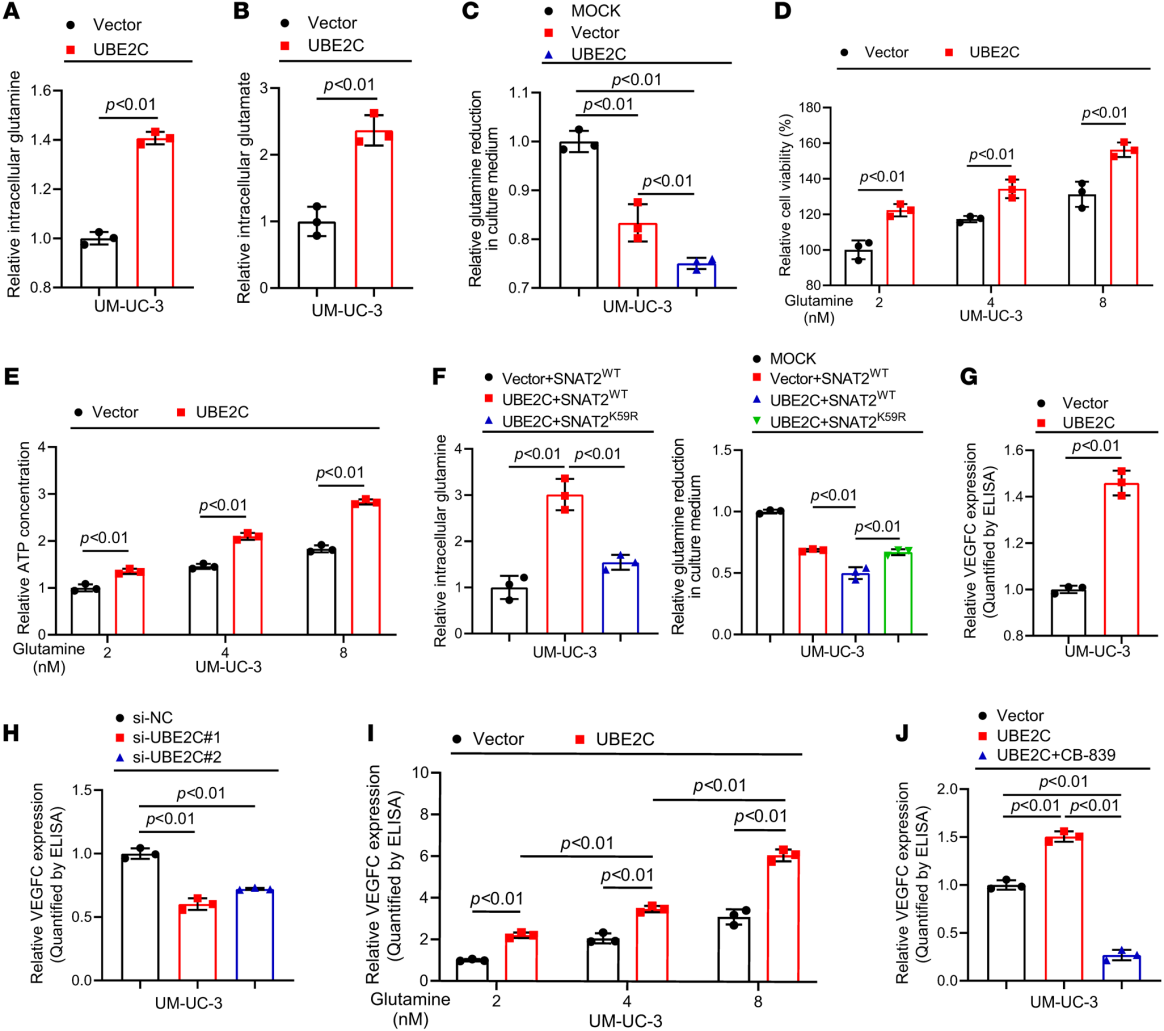
K59 monoubiquitination of SNAT2 promotes the secretion of VEGFC by increasing glutamine uptake and metabolism. (**A** and **B**) Detection of glutamine and glutamate in the cell extracts for analysis of glutamine uptake by UBE2C-overexpressing UM-UC-3 cells. (**C**) Detection of culture medium glutamine reduction in UBE2C-overexpressing UM-UC-3 cells. (**D**) Cell Counting Kit-8 analysis of cell viability in UBE2C-overexpressing UM-UC-3 cells. (**E**) ATP assay kit analysis of intracellular ATP production in UBE2C-overexpressing UM-UC-3 cells. (**F**) Detection of glutamine uptake in UM-UC-3 cells after SNAT2^K59R^ mutation. (**G** and **H**) ELISAs of VEGFC secretion in UBE2C-overexpressing or -knockdown UM-UC-3 cells. (**I** and **J**) ELISAs of VEGFC secretion after the addition of exogenous glutamine or CB-839, a glutamine metabolism inhibitor, to UBE2C-overexpressing UM-UC-3 cells. Significant differences were identified through 1-way ANOVA followed by Dunnett’s test (**C**, **F**, **H**, **I**, and **J**) and 2-tailed Student’s *t* test (**A**, **B**, **D**, **E**, and **G).** Quantitative results are presented as the mean ± SEM of 3 separate experiments.

SNAT2 plays an essential role in glutamine transport (36). Therefore, we investigated the possible function of UBE2C in glutamine metabolism. Glutamine and glutamate assays showed that, compared with levels in the control group, glutamine and glutamate levels in the cellular extracts were increased (Figure 5, A and B, and Supplemental Figure 7, A and B), and glutamine levels in the culture medium were reduced (Figure 5C and Supplemental Figure 7C). Moreover, overexpression of UBE2C increased cell viability and ATP production (Figure 5, D and E, and Supplemental Figure 7, D and E), indicating that overexpression of UBE2C promoted glutamine uptake and glutamine metabolism. However, the SNAT2^K59R^ mutation abolished the increase in glutamine uptake and metabolism mediated by UBE2C overexpression (Figure 5F and Supplemental Figure 7, F–H), demonstrating that UBE2C promoted glutamine metabolism through ubiquitination of SNAT2 at K59.

Our previous reports revealed that VEGFC is the key regulator of tumor lymphangiogenesis (37). Therefore, we next investigated the effect of glutamine metabolism on the secretion of VEGFC. ELISAs and Western blotting revealed that overexpression of UBE2C substantially promoted VEGFC secretion (Figure 5G and Supplemental Figure 7, I and J), whereas knockdown of UBE2C had the opposite effect (Figure 5H and Supplemental Figure 7, K and L). Furthermore, experiments with addition of exogenous glutamine indicated that increased glutamine metabolism facilitated the secretion of VEGFC from BCa cells (Figure 5I and Supplemental Figure 7, M and N). Moreover, the increase in VEGFC secretion induced by overexpression of UBE2C was significantly attenuated after treatment with a glutamine metabolic inhibitor (CB-839) (Figure 5J and Supplemental Figure 7, O and P), indicating that increased glutamine metabolism stimulated VEGFC secretion. Collectively, these findings suggest that membrane-enriched SNAT2 facilitated VEGFC secretion through increases in glutamine uptake and glutamine metabolism.

### UBE2C facilitates BCa lymphangiogenesis and LN metastasis via VEGFC.

**Figure 6:**
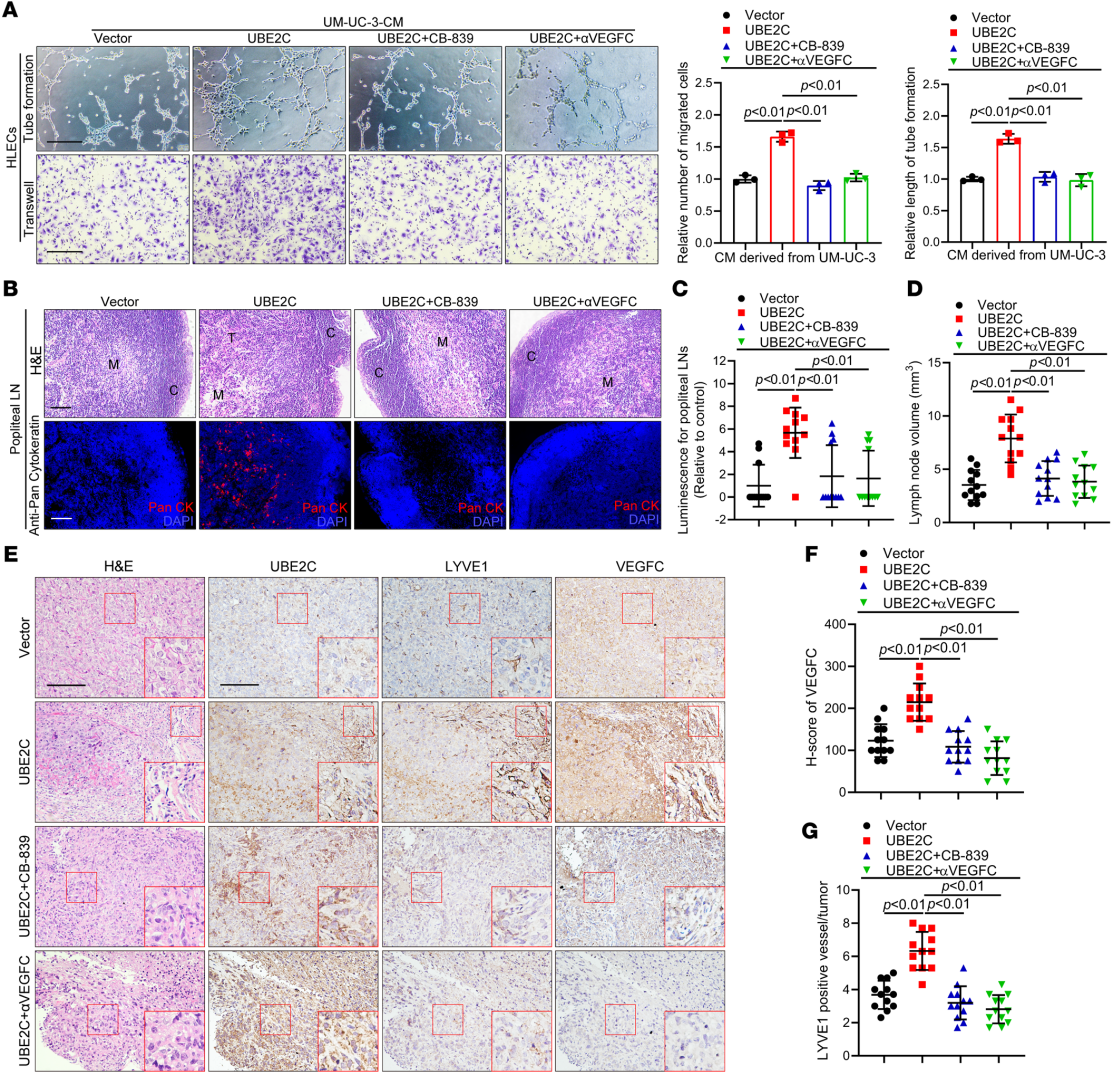
Inhibition of glutamine metabolism or VEGFC secretion suppresses lymphangiogenesis and LN metastasis. (**A**) Representative images and quantification of tube formation and migration of HLECs treated with culture media from UBE2C-overexpressing UM-UC-3 cells with or without CB-839 and anti-VEGFC (αVEGFC) treatment. Scale bars: 100 μm. (**B**) Representative fluorescence images of popliteal LNs from mice treated with or without CB-839 or anti-VEGFC (*n* = 12). Scale bars: 100 μm. (**C** and **D**) Quantification of affected popliteal LNs from the mice treated with or without CB-839 and anti-VEGFC (*n* = 12). (**E**–**G**) Representative IHC images and quantification of VEGFC expression and LYVE1-indicated MLD in primary footpad tumor tissues from the mice treated with or without CB-839 and anti-VEGFC (*n* = 12). Scale bars: 50 μm. Original magnification, ×2 (enlarged insets). Significant differences were identified through 1-way ANOVA followed by Dunnett’s test (**A**, **C**, **D**, **F**, and **G**). The quantitative results are presented as the mean ± SEM of 3 separate experiments.

We then performed in vitro and in vivo experiments to determine whether UBE2C facilitates BCa lymphangiogenesis and LN metastasis by promoting glutamine metabolism and VEGFC secretion. In vitro experiments revealed that the increase in the tube formation and migration of HLECs promoted by UBE2C overexpression was abolished by CB-839 or anti-VEGFC antibody (a VEGFC-neutralizing antibody) treatment (Figure 6A and Supplemental Figure 8, A–C). Treatment with CB-839 or anti-VEGFC significantly inhibited the metastasis of UBE2C-overexpressing BCa cells from the footpad to the popliteal LNs (Figure 6, B–D, and Supplemental Table 5). IHC analysis revealed that, compared with expression levels in the control group, the group with upregulated UBE2C exhibited markedly increased MLD and VEGFC expression in mouse footpad tumors, while treatment with CB-839 or anti-VEGFC dramatically suppressed these effects (Figure 6, E–G). Collectively, these findings confirm that UBE2C facilitated lymphangiogenesis and LN metastasis in BCa by promoting the secretion of VEGFC.

### Therapeutic efficacy of UBE2C in a patient-derived xenograft model of LN-metastatic BCa.

**Figure 7:**
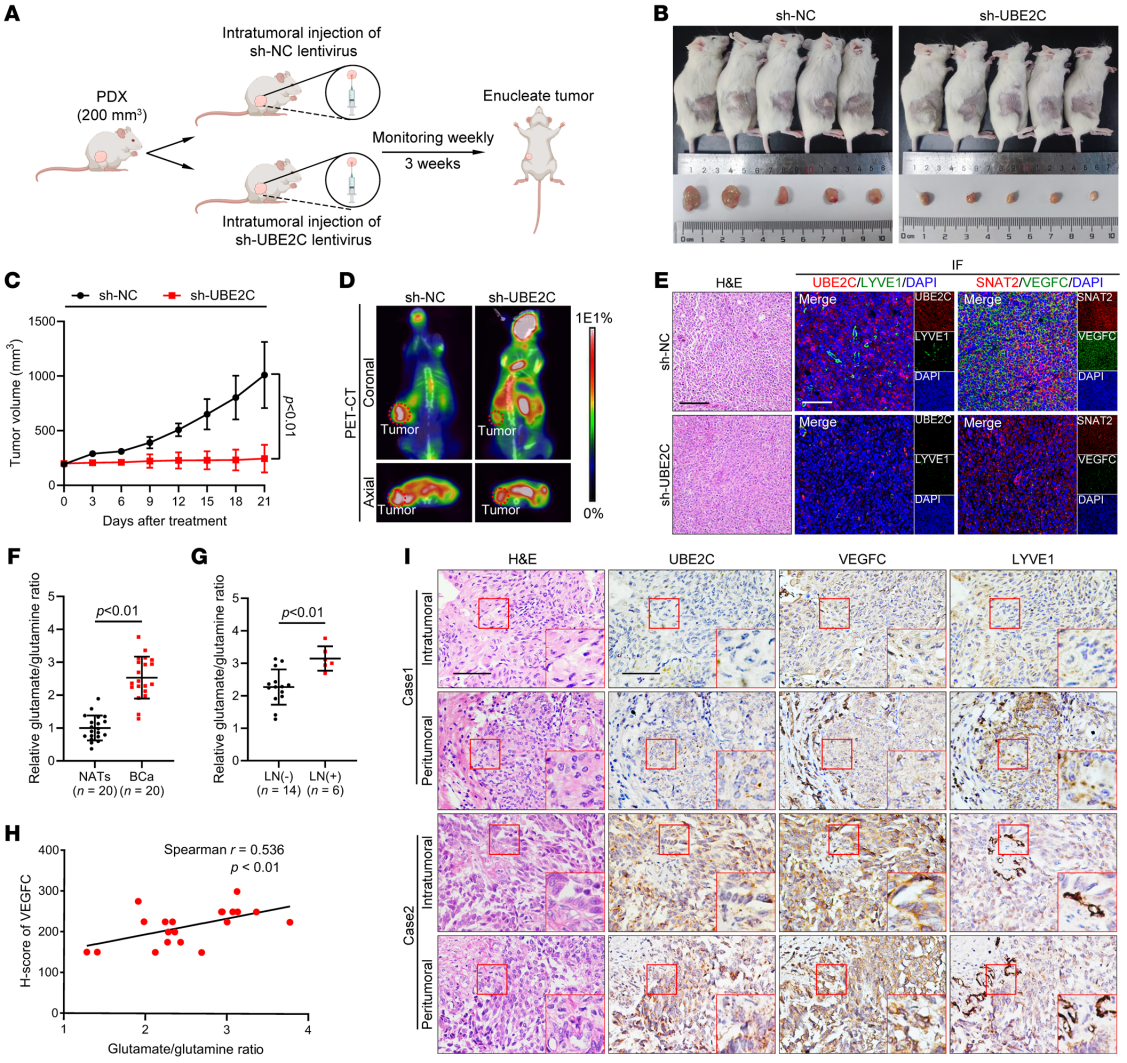
Inhibition of UBE2C suppresses the growth of PDX tumors from LN-metastatic BCa. (**A**) Schematic illustration of the procedure for constructing the PDX model. (**B** and **C**) Images and quantification of tumor volume in the mice treated with sh-UBE2C or sh-NC (*n* = 5). (**D**) Representative PET-CT images of tumors from the mice treated with sh-UBE2C or sh-NC. (**E**) Representative images of IF staining showing UBE2C, SNAT2 and VEGFC expression and LYVE1-indicated microlymphatic vessel density in tumor tissues from PDXs. Scale bars: 50 μm. (**F**) Detection of the glutamate/glutamine ratio in paired BCa tissues and NATs (*n* = 20). (**G**) Detection of the glutamate/glutamine ratio in BCa tissues with or without LN metastasis. (**H**) Correlation analysis of the glutamate/glutamine ratio and the expression of VEGFC in BCa tissues (*n* = 20). (**I**) Representative IHC images of UBE2C, VEGFC, and LYVE1-indicated MLD in both intratumoral and peritumoral regions of BCa tissues (*n* = 323). Scale bars: 50 μm. Original magnification, ×2 (enlarged insets). Significant differences were identified through 2-tailed Student’s *t* test (**C**, **F**, and **G**). The quantitative results are presented as the mean ± SEM of 3 separate experiments.

Given the critical role that UBE2C plays in controlling LN metastasis in BCa, the therapeutic effect of UBE2C inhibition was further investigated in a patient-derived xenograft (PDX) model of LN-metastatic BCa tissue. When the volume of the PDX tumors reached approximately 200 mm^3^, the mice were randomly allocated into 2 groups and received intratumoral injections of sh-UBE2C or sh-NC (Figure 7A). The PDX models showed that treatment with sh-UBE2C resulted in significant reductions in tumor volume compared with tumor volumes in the control group (Figure 7, B–D), indicating that silencing UBE2C had a therapeutic effect on the progression of BCa. Furthermore, IF staining showed that knockdown of UBE2C significantly reduced the secretion of VEGFC and the MLD in mouse tumors (Figure 7E and Supplemental Figure 8, D–H). Taken together, these results suggest that silencing UBE2C inhibited BCa growth and lymphangiogenesis and that targeting UBE2C is a promising therapeutic strategy for preventing LN metastasis in patients with BCa.

### Clinical relevance of the UBE2C/SNAT2/VEGFC axis in patients with BCa.

We further investigated the clinical relevance of the UBE2C/SNAT2/VEGFC regulatory axis. We performed glutamine and glutamate assays as well as IHC on 20 paired BCa tissues and NATs to evaluate the glutamate/glutamine ratio and VEGFC expression. Compared with the NATs, the BCa tissues exhibited a significantly elevated glutamate/glutamine ratio (Figure 7F). Furthermore, a greater glutamate/glutamine ratio was detected in the BCa tissues with LN metastasis than in the BCa tissues without metastasis (Figure 7G). Moreover, correlation analysis revealed that the glutamate/glutamine ratio was positively associated with the expression of VEGFC (Figure 7H). IHC analysis of BCa tissues revealed a significant correlation between elevated UBE2C expression and increased VEGFC expression and MLD (Figure 7I and Supplemental Figure 8, I and J). Collectively, our findings demonstrate that the UBE2C/SNAT2/VEGFC axis is crucial in BCa lymphangiogenesis and LN metastasis.

## Discussion

Crosstalk between different types of ubiquitination alters the biological outcomes of proteins, thereby facilitating tumor development (17). However, the involvement of ubiquitination-type crosstalk in LN metastasis and the pivotal regulatory factors governing this process have not been determined. In this study, we identified a functional role of UBE2C in inducing crosstalk between the monoubiquitinated and polyubiquitinated forms of SNAT2 to promote lymphangiogenesis and LN metastasis in patients with BCa. UBE2C interacts with SNAT2 to promote the monoubiquitination of SNAT2 at the K59 residue, which inhibits the elongation of the K63-linked polyubiquitin chain of SNAT2 at the K33 residue by blocking the interaction between SNAT2 and NEDD4L. The crosstalk between the monoubiquitinated and polyubiquitinated forms alters the subcellular localization of SNAT2 and promotes BCa lymphangiogenesis and LN metastasis. These results highlight a function of UBE2C in mediating crosstalk between monoubiquitination and polyubiquitination of SNAT2 to promote BCa lymphangiogenesis and LN metastasis and indicate that UBE2C is a promising therapeutic target for LN-metastatic BCa.

Ubiquitination orchestrates the life cycle of membrane proteins (38). On the one hand, ubiquitination facilitates the internalization and degradation of membrane proteins (39); on the other hand, it regulates the translocation of newly synthesized mature proteins to the membrane (40). Here, we found that crosstalk between the monoubiquitinated and polyubiquitinated forms increased the membrane expression level of SNAT2. This crosstalk modulated the interaction between the substrate and ubiquitin-binding protein EPN1 by altering the length of the ubiquitin chain, thereby inhibiting the endocytosis of ubiquitinated proteins. Our results reveal a molecular mechanism for ubiquitination-mediated membrane protein accumulation and suggest that E2s may serve as potential targets for the clinical treatment of tumors involving membrane proteins.

Glutamine, which is primarily absorbed by cancer cells, is crucial for metabolism, as it provides energy to cancer cells to accelerate growth and proliferation (41). Accumulating evidence shows that glutamine metabolism also plays an important role in tumor invasion (42), but the precise role and molecular mechanisms involved in the microenvironment of lymphatic metastasis have not been determined. Here, we showed that the glutamate/glutamine ratio was greater in BCa tissues from patients with LN metastasis than in those from patients without metastasis. The glutamate/glutamine ratio was positively correlated with the concentration of VEGFC in BCa tissues. Increased glutamine metabolism promoted lymphangiogenesis and LN metastasis in BCa by facilitating the secretion of VEGFC. Moreover, we confirmed that inhibition of glutamine metabolism with CB-839 effectively suppressed BCa lymphangiogenesis and popliteal LN metastasis in vivo and in vitro. These results reveal the pivotal correlation between glutamine metabolism and lymphangiogenesis and deepen our understanding of glutamine reprogramming in LN metastasis in patients with BCa.

Immunotherapy has emerged as a promising avenue for treating patients with cancer (43). However, the objective response rate for immunotherapy in patients with BCa is only approximately 20% (44–46). Numerous studies have revealed that LN metastasis is a systemic disease that rewires the entire immune system and impairs the efficacy of immunotherapy (1, 47). Therefore, inhibiting the LN metastasis of tumors has dual purposes: preventing distant metastasis and rejuvenating antitumor immune responses. Here, we showed that *UBE2C* was more highly expressed in BCa tissues than in NATs and confirmed that *UBE2C* was positively related to the lymphangiogenesis and LN metastasis of patients with BCa in a large cohort. Moreover, we established PDX models using LN-metastatic BCa tissues for therapeutic experiments, in which UBE2C lentivirus treatment markedly suppressed tumor growth and lymphangiogenesis. Our results indicate that targeting UBE2C to inhibit BCa lymphangiogenesis and LN metastasis offers a possible treatment approach for BCa with LN metastasis.

In summary, our findings elucidate a mechanism by which UBE2C activates the crosstalk between the monoubiquitinated and polyubiquitinated forms of SNAT2, thereby facilitating the accumulation of SNAT2 at the plasma membrane and increasing glutamine uptake and metabolism to promote lymphangiogenesis in BCa and facilitate LN metastasis. This systematic study revealed the role of UBE2C in inducing crosstalk between the monoubiquitinated and polyubiquitinated forms of SNAT2 that promotes LN metastasis in patients with BCa, suggesting that UBE2C is a potential target for the treatment of LN-metastatic BCa.

## Methods

### Sex as a biological variable.

Male and female human BCa samples were analyzed. Female mice were used in all mouse studies. In this study, sex was not considered as a biological variable.

### Supplemental material.

Additional details on methods are available in the Supplemental Methods.

### Patients and clinical samples.

At Sun Yat-sen University, affiliated with Sun Yat-sen Memorial Hospital (Guangzhou, China), 323 pairs of NATs and BCa tissues were collected from patients (*n* = 42 women; *n* = 281 men) undergoing surgical resection. For every clinical sample, the histological and pathological types were independently determined by 2 skilled pathologists. The clinical and pathological data can be found in Supplemental Table 1.

### Cell lines and cell culturing.

The American Type Culture Collection (ATCC) was the source of the human BCa cell lines 5637 (catalog HTB-9, RRID: CVCL_0126), UM-UC-3 cells (catalog CRL-1749, RRID: CVCL_1783), and T24 cells (catalog HTB-9, RRID: CVCL_0554), as well as the SV-HUC-1 cells (catalog CRL-9520, RRID: CVCL_3798). ScienCell Research Laboratories provided the HLECs. RPMI 1640 medium (Gibco, Thermo Fisher Scientific) was used to culture T24 and 5637 cells. DMEM (Gibco, Thermo Fisher Scientific) was used for UM-UC-3 cells. Endothelial cell medium (ScienCell) was used for HLEC cultures, and Ham’s F12K medium (Gibco, Thermo Fisher Scientific) was used for SV-HUC-1 cell cultures. All media were supplemented with 10% FBS (Gibco, Thermo Fisher Scientific), except for the HLEC medium, which was supplemented with 5% FBS and 1% corresponding growth factor (ScienCell Research Laboratories). All cell lines were cultured in a 37°C humid atmosphere of 5% CO_2_.

### Lentivirus infection and cell transfection.

The plasmids, siRNAs, and lentiviruses were purchased from Igebio. The plasmids and siRNAs were transfected in the presence of Lipofectamine 3000 (Invitrogen, Thermo Fisher Scientific, catalog L3000075) according to the manufacturer’s protocols. For lentivirus infection, BCa cells were infected with lentivirus and screened with puromycin (Selleck Chemicals, catalog S7417) for 2 weeks. Transfection efficiency was assessed by qRT-PCR and Western blotting.

### Popliteal lymphatic metastasis model.

A total of 24 female 4-week-old BALB/c nude mice (weight, 18–20 g) were purchased from Guangdong Yaokang Biotechnology Co. Ltd. and housed at the Experimental Animal Center of Sun Yat-sen University. Subsequently, mouse footpads were injected with a 25 μL suspension containing approximately 5 × 10^5^ luciferase-expressing mCherry UM-UC-3 cells with or without overexpression of UBE2C. Popliteal lymphatic metastasis was imaged weekly with an IVIS (Xenogen). When the tumor volume reached nearly 200 mm^3^, the mice were overanesthetized with pentobarbital, and the footpad tumors and popliteal LNs were resected and paraffin embedded for IHC or IF analysis.

### Establishment and treatment of PDXs.

Four-week-old female NOD/SCID/IL2rg-null (NSG) mice received subcutaneous implants of fresh BCa tissues obtained from surgically treated patients (first generation, F1). The tumors were divided into pieces and inserted into F2 mice once the xenografts reached a size of 400 mm^3^. After this, the F3 mice were implanted with tumor tissues of equal size obtained from the F2 mice. When the F3 tumor volume reached 200 mm^3^, the mice were randomly divided into 2 groups (*n* = 5 per group), and the control lentivirus (sh-NC) or the in vivo–optimized UBE2C silencing lentivirus (sh-UBE2C) was injected into the tumor. Tumor volume was measured twice per week. After 21 days, the tumors were resected and paraffin embedded for IHC or IF analysis. Pentobarbital anesthesia was administered before all the procedures and examinations.

### Co-IP assay.

Cells were harvested and lysed in IP lysis buffer (Thermo Fisher Scientific, catalog 87787) supplemented with protease inhibitors (Thermo Fisher Scientific, catalog 78427) and phosphatase inhibitors (Thermo Fisher Scientific, catalog 87786). The cells were then fully lysed for 30 minutes on ice. Subsequently, the mixture was centrifuged at 4°C and 16,000*g* for 20 minutes to obtain the lysate supernatant. After overnight incubation at 4°C with primary antibodies, the supernatants were incubated for 3 hours with protein A/G beads (Thermo Fisher Scientific, catalog 88803). Thereafter, the protein A/G beads were washed 3 times with lysis buffer and subjected to MS analysis or eluted for analysis by western blotting.

### Measurement of glutamine uptake and glutamate production.

Glutamine uptake and glutamate production were measured with a glutamine assay kit (Chemical Book, catalog ADS-W-N003-48) and a glutamate assay kit (Chemical Book, catalog ADS-W-AJS007) according to the manufacturer’s instructions. Glutamine uptake by cells was calculated by subtracting the measured glutamine concentration in the medium from the original glutamine concentration. All values were normalized to the cell number or tissue weight.

### Statistics.

The quantitative results are presented as the mean ± SEM of 3 separate experiments. If the data were normally distributed, 1-way ANOVA followed by Dunnett’s test or the 2-tailed Student’s *t* test was used to assess mean differences. The H-score was used to evaluate the statistical significance of the differences detected via IHC analysis. The χ^2^ test was used to assess the clinicopathological significance of clinical samples in cases of categorical data. K-M analysis and the log-rank test (Mantel-Cox) were used for survival analysis. In all the statistical analyses, a *P* value less than 0.05 indicated statistical significance.

### Study approval.

Written informed consent was obtained from all patients. This study received the approval of the Committees for Ethics Review of Research involving Human Subjects at Sun Yat-sen University (approval no. SYSEC-KY-KS-2021-392). Clinical data and sample collection were authorized by the ethics committee of Sun Yat-sen Memorial Hospital, Sun Yat-sen University. The research was conducted in compliance with accepted ethics standards. The animal studies were performed after receiving approval from the IACUC of Sun Yat-sen University (approval no. AP20230217). The construction of the popliteal lymphatic metastasis model was conducted in accordance with the guidelines set by the institution, and the model was approved by the IACUC of Sun Yat-sen University.

### Data availability.

All data from the present study are available in the Supporting Data Values file or from the corresponding author. The sequencing data generated in this study are publicly available in the NCBI GEO database (GEO GSE106534).

## Author contributions

WL wrote the original draft, handled project administration, and the in vitro and in vivo experiments. CC participated in the study design. HZ and YL conducted the data analyses. MA and DL performed the clinical data analyses. YZ participated in the revision of the manuscript and supplemental experiments. MG and TL performed the IF and IHC experiments. WH designed the study and wrote the manuscript. All authors have read and approved the final manuscript. The authorship order among the co–first authors was determined on the basis of their relative contributions.

## Supplementary Material

Supplemental data

Unedited blot and gel images

Supporting data values

## Version 1 07/01/2024

Electronic publication
