# Supplementary material for: UBE2C-induced crosstalk between mono- and polyubiquitination of SNAT2 promotes lymphatic metastasis in bladder cancer
Source: J Clin Invest. 2024 Jul 1;134(13):e179122. doi: 10.1172/JCI179122 (PMC11213464; doi:10.1172/JCI179122)
Supplement: Supplemental data [file jci-134-179122-s217.pdf]

Supplemental Figures

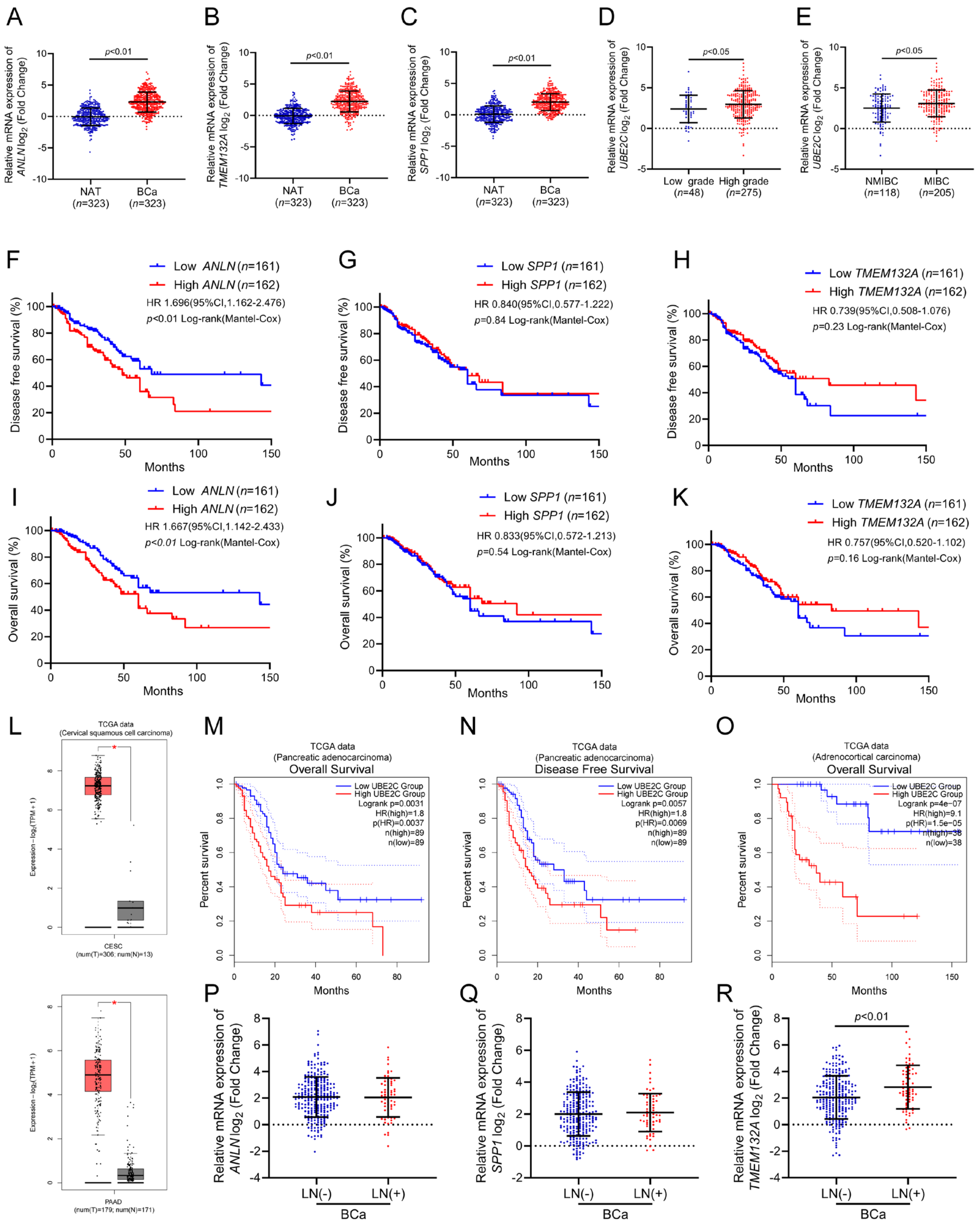

Supplemental Figure 1. *UBE2C* is overexpressed in various human cancers and is correlated with a poor patient prognosis. (**A-C**) qRT-PCR analysis of *ANLN, TMEM132A, SPP1* expression in BCa tissues versus NATs (n = 323). (**D**) qRT-PCR analysis of *UBE2C*

expression in high-grade BCa versus low-grade BCa (n = 323). (**E**) qRT-PCR analysis of *UBE2C* expression in MIBC versus NMIBC. (**F-K**) K-M survival analysis of the disease-free survival and overall survival of patients with BCa with low versus high *ANLN, SPP1* and *TMEM132A* expression. The cutoff value is the median. (**L**) Analysis of the TCGA database revealed *UBE2C* expression in various human cancers compared with that in normal tissue. (**M-O**) K-M survival analysis of patients grouped according to *UBE2C* expression for different cancer types in the TCGA database. (**P-R**) qRT-PCR analysis of *ANLN, SPP1* and *TMEM132A* expression in LN-positive BCa versus LN-negative BCa (n = 323). The statistical significance of differences was assessed through the nonparametric Mann-Whitney U test in **A-E, L** and **P-R**. Data are shown as the mean ± SEM.

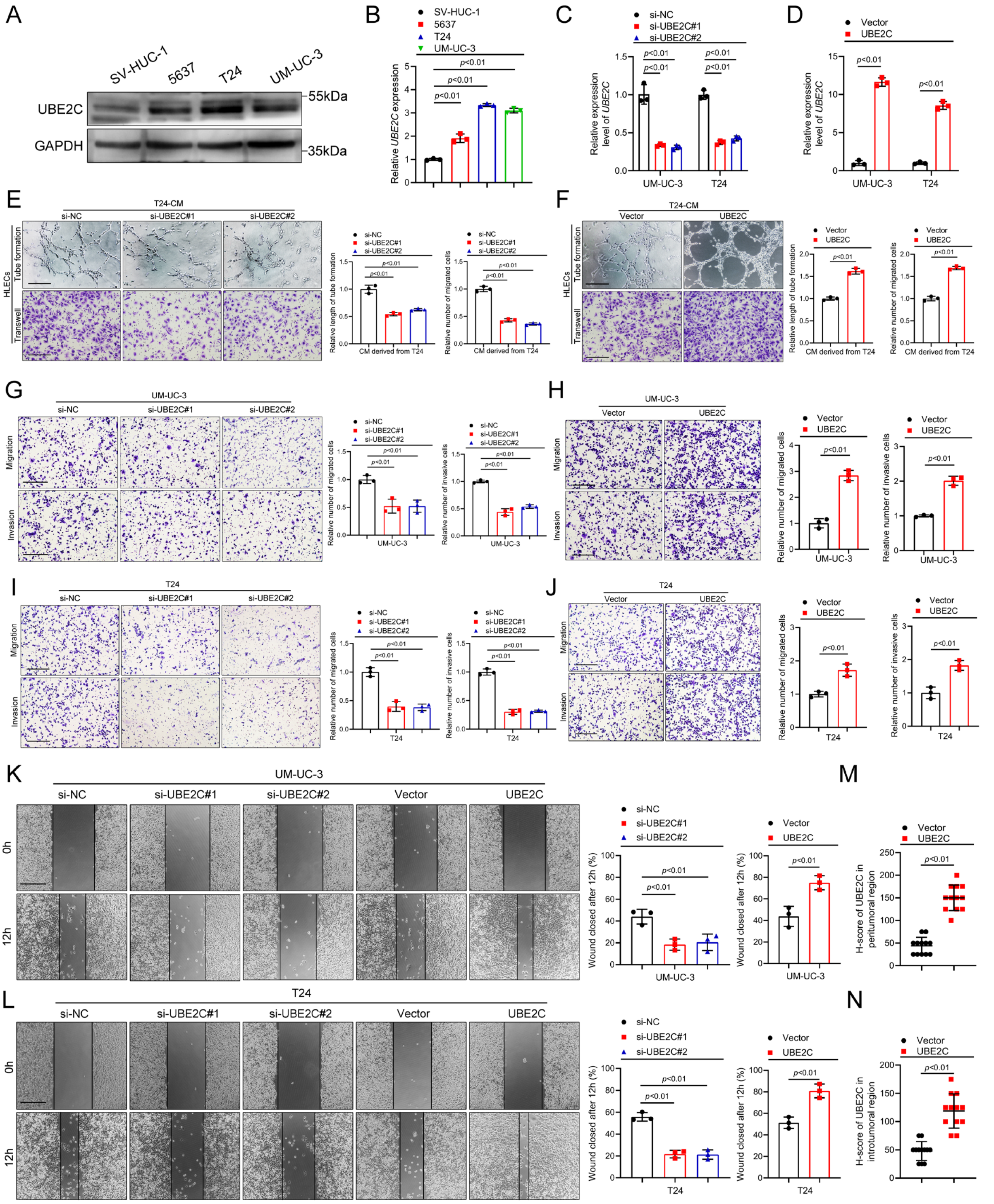

Supplemental Figure 2. UBE2C promotes the invasion and migration of BCa cells in vitro. (**A and B**) Western blotting analysis (**A**) and qRT-PCR analysis (**B**) of the expression level of UBE2C in 5637, T24, UM-UC-3 and SV-HUC-1 cells. (**C and D**) qRT-PCR analysis

of UBE2C expression following UBE2C overexpression or knockdown in BCa cells. (**E-F**) Representative images and quantification of the tube formation and migration of HLECs after coculture with UBE2C knockdown or UBE2C-overexpressing T24 cells. Scale bars: 100 μm. (**G-J**) Representative images and quantification of the migration and invasion of BCa cells after downregulating or overexpressing UBE2C. Scale bars: 100 μm. (**K and L**) Representative images and quantification of wound healing assays showing the migration capability of UM-UC-3 and T24 cells after downregulation or overexpression of UBE2C. Scale bars: 100 μm. (**M and N**) Quantification of UBE2C expression in the peritumoral (**M**) and intratumoral (**N**) regions of footpad tumor tissues. Significant differences were identified through 1-way ANOVA followed by Dunnett's test in **B, C, E, G, I, K** and **L**; 2-tailed Student's t test in **D, F, H, J, M** and **N**. Data are shown as the mean ± SEM.

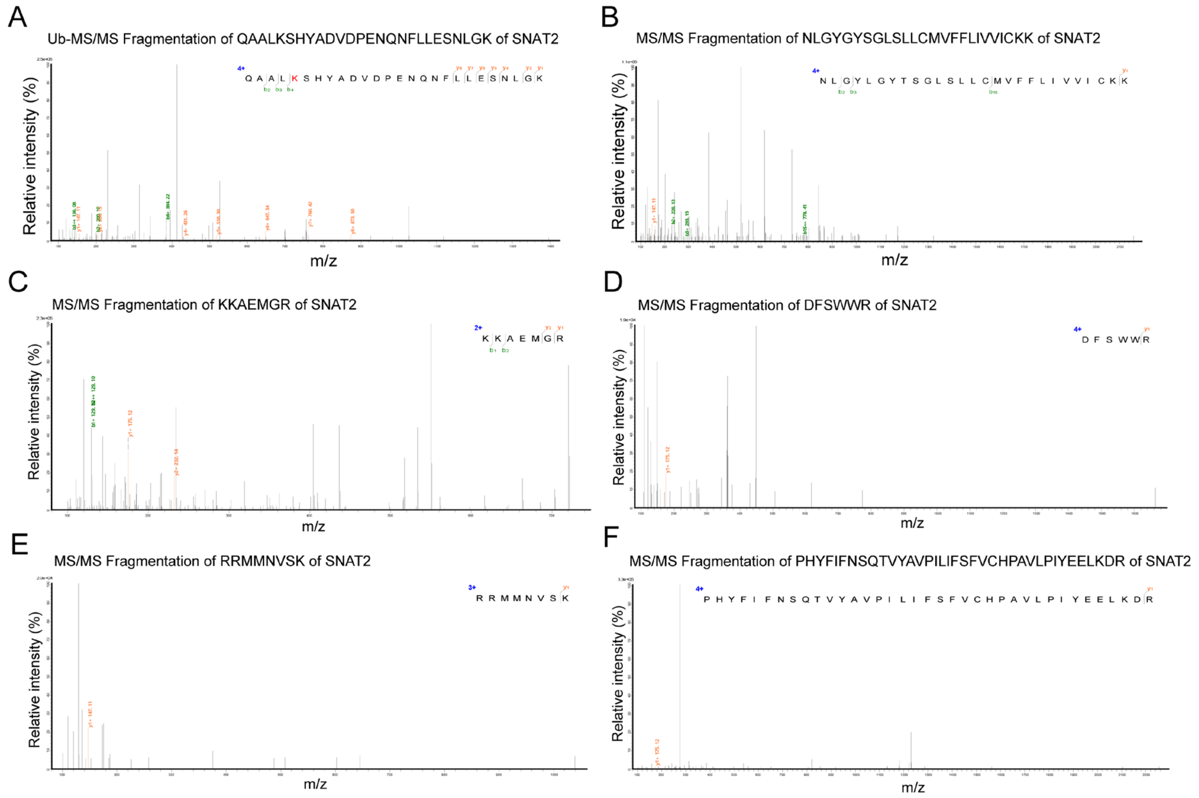

Supplemental Figure 3. SNAT2 interacts with UBE2C. (**A**) Mass spectrometry analysis for the detection of UBE2C ubiquitinating proteins. (**B-F**) Mass spectrometry analysis of UBE2C-binding proteins after co-IP assays.

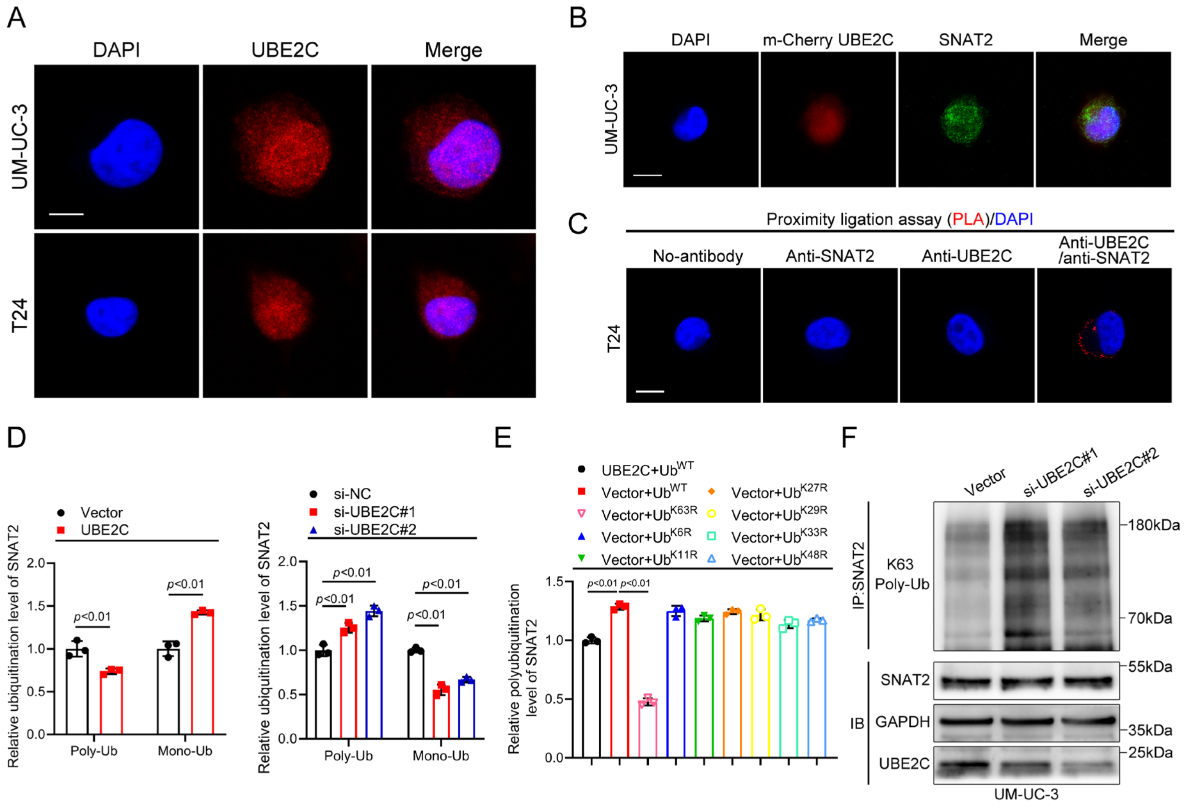

Supplemental Figure 4. UBE2C inhibits K63-linked polyubiquitination of SNAT2. (**A**) Detection of intracellular localization of UBE2C in BCa cells. Scale bars: 5 µm. (**B**) Immunofluorescence assays showing the colocalization of SNAT2 and UBE2C. Scale bars: 5 µm. (**C**) Proximity ligation assays showing the interaction between UBE2C and SNAT2. Scale bars: 5 µm. (**D**) Quantification of the mono- and polyubiquitination levels of SNAT2 after overexpression or knockdown of UBE2C. (**E**) Quantification of the polyubiquitination levels of SNAT2 after mutation of ubiquitin. (**F**) IB analysis of the K63-linked polyubiquitination level of SNAT2 after si-UBE2C. Significant differences were identified through 2-tailed Student's t test in **D**; 1-way ANOVA followed by Dunnett's test in **D, E.** Data are shown as the mean ± SEM.

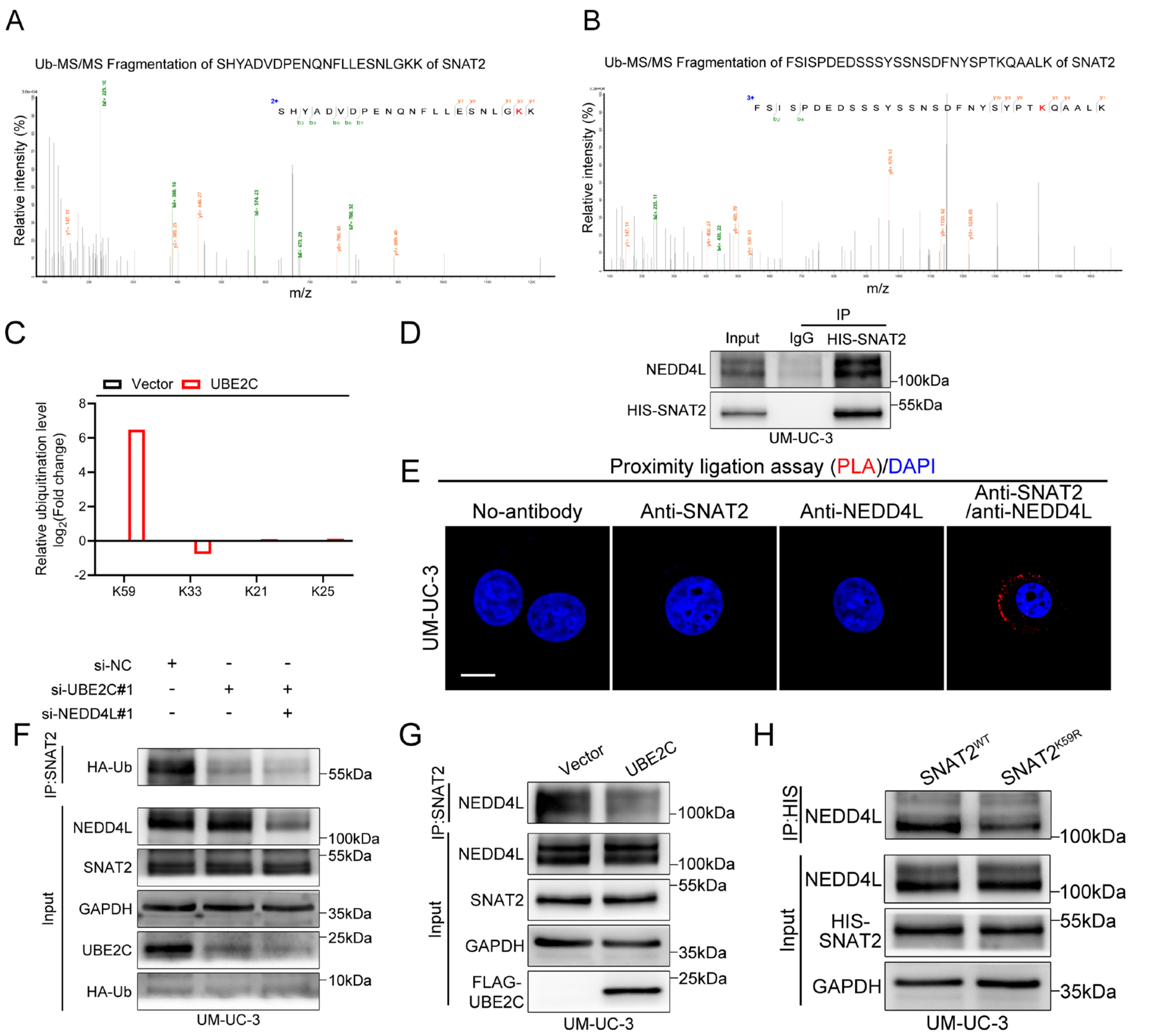

Supplemental Figure 5. UBE2C blocks the interaction between SNAT2 and NEDD4L. (**A**) Mass spectrometry analysis of the UBE2C-mediated ubiquitination site in SNAT2. (**B**) Mass spectrometry analysis of UBE2C-inhibited ubiquitination site in SNAT2. (**C**) Quantification of ubiquitination level of various lysine residues within SNAT2. (**D**) IB analysis after co-IP assays with anti-HIS or IgG in UM-UC-3 cells. (**E**) Proximity ligation assays showing the interaction between SNAT2 and NEDD4L. Scale bars: 5 µm. (**F**) IB analysis of the monoubiquitination level of SNAT2 after si-UBE2C and si-NEDD4L transfection. (**G**) IB analysis of the interaction between NEDD4L and SNAT2 after UBE2C

overexpression. (**H**) IB analysis of the interaction between NEDD4L and SNAT2 after $SNAT2^{K59R}$ mutation.

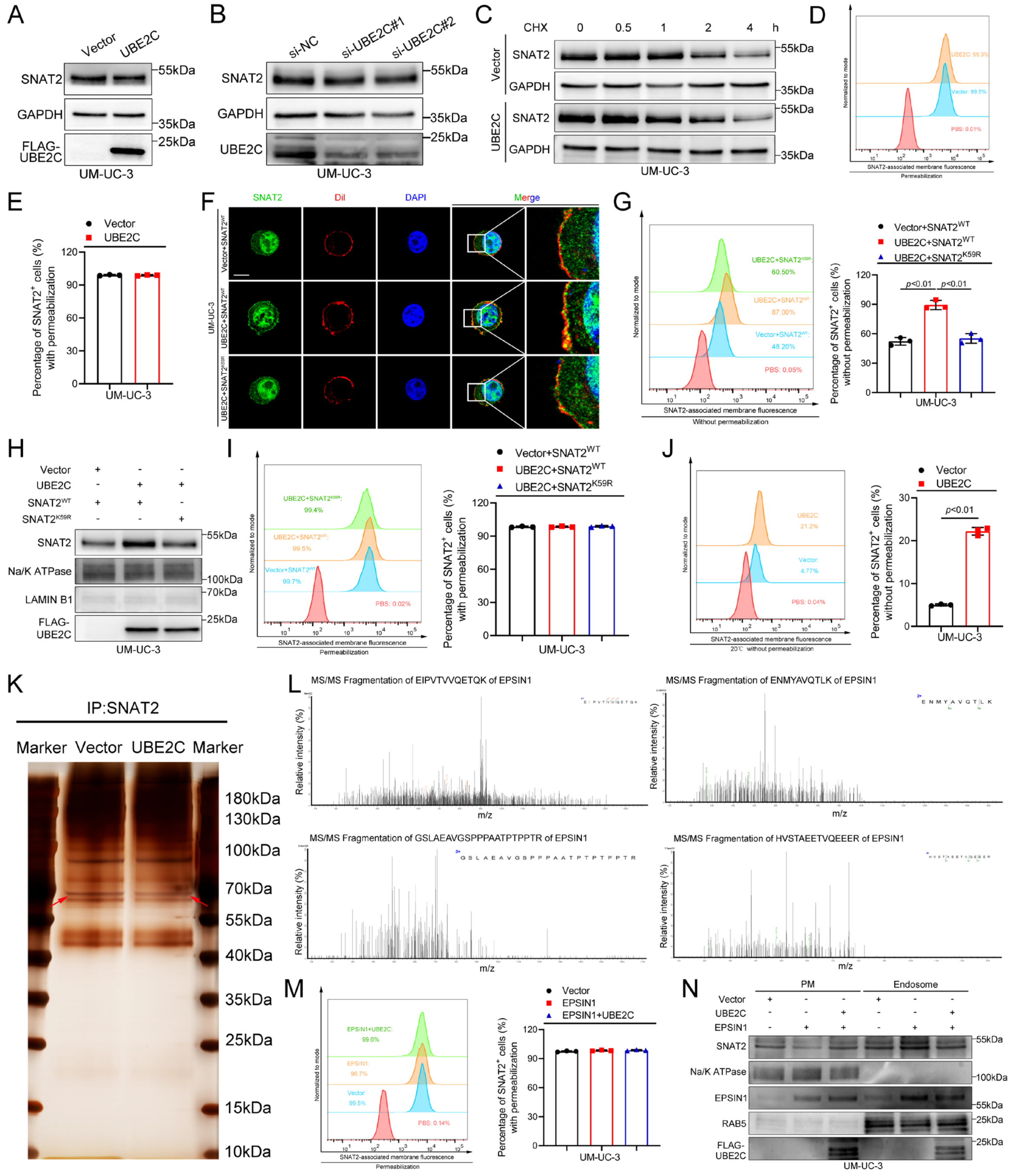

Supplemental Figure 6. UBE2C inhibits the endocytosis of SNAT2. (**A**) IB analysis of SNAT2 expression in BCa cells overexpressing UBE2C. (**B**) IB analysis of SNAT2 expression in BCa cells after downregulating UBE2C. (**C**) IB analysis of the half-life of SNAT2 after overexpression of UBE2C. (**D and E**) FACS analysis with permeabilization and quantification of SNAT2 expression after overexpressing UBE2C. **(F-H)** Detection of SNAT2

expression in membrane fractions after SNAT2^{K59R} mutation in UM-UC-3 cells. Scale bar: 5 μm. (**I**) FACS analysis of the permeabilization and quantification of SNAT2 expression after SNAT2^{K59R} mutation. (**J**) FACS analysis with quantification of SNAT2 expression in membrane fractions after overexpressing UBE2C at 20°C. (**K**) Silver staining for the detection of SNAT2-interacting proteins. (**L**) Mass spectrometry analysis of SNAT2-binding proteins after co-IP assays. (**M**) FACS analysis with permeabilization and quantification of SNAT2 expression after overexpressing EPSIN1 and UBE2C. (**N**) IB analysis of SNAT2 expression in membrane and endosome fractions after overexpression of UBE2C and EPSIN1. Significant differences were identified through 1-way ANOVA followed by Dunnett's test in **G, I** and **M**; and 2-tailed Student's t test in **E** and **J**. Data are shown as the mean ± SEM.

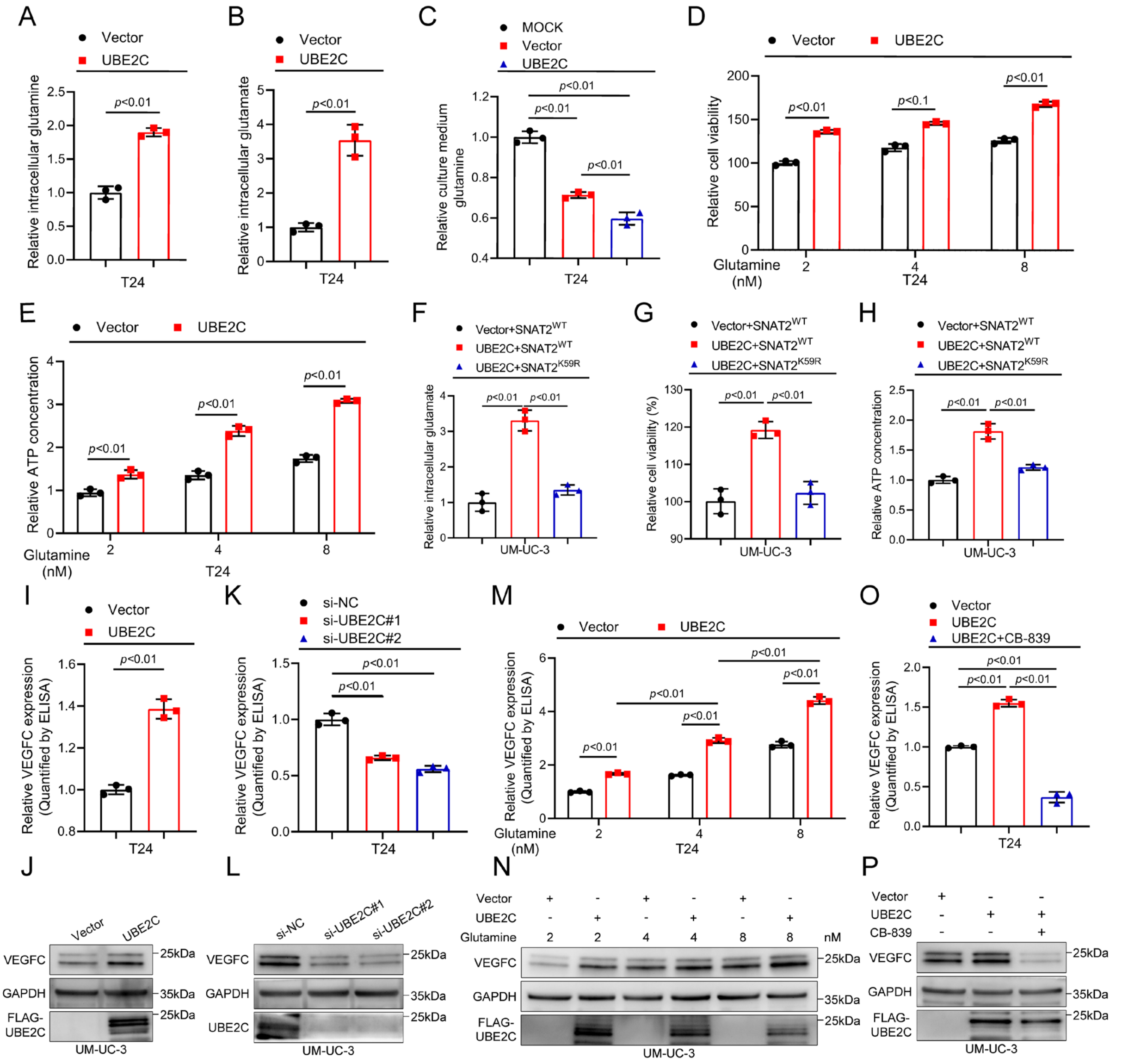

Supplemental Figure 7. UBE2C activates glutamine reprogramming to promote VEGFC secretion. (**A and B**) Detection of glutamine and glutamate production in the cell extracts for analysis of glutamine uptake by UBE2C-overexpressing T24 cells. (**C**) Analysis of culture media glutamine in UBE2C-overexpressing T24 cells with a glutamine assay kit. (**D**) CCK-8 analysis of cell viability in UBE2C-overexpressing T24 cells. (**E**) Analysis of intracellular ATP production in UBE2C-overexpressing T24 cells with an ATP assay kit. (**F-H**) Detection of glutamate, cell viability and ATP production in UM-UC-3 cells after $SNAT2^{K59R}$

mutation. (**I**) ELISA analysis of VEGFC secretion in T24 cells with overexpression of UBE2C. (**J**) IB analysis of VEGFC secretion in UM-UC-3 cells with overexpression of UBE2C. (**K**) ELISA analysis of VEGFC secretion in T24 cells with knockdown of UBE2C. (**L**) IB analysis of VEGFC secretion in UM-UC-3 cells with knockdown of UBE2C. (**M**) ELISA analysis of VEGFC secretion after the addition of exogenous glutamine. (**N**) IB analysis of VEGFC secretion after the addition of exogenous glutamine. (**O**) ELISA analysis of VEGF-C secretion after the addition of glutamine metabolism inhibitors. (**P**) IB analysis of VEGF-C secretion after the addition of glutamine metabolism inhibitors. Significant differences were identified through 1-way ANOVA followed by Dunnett's test in **C, F, G, H, K, M and O**; 2-tailed Student's t test in **A**, **B, D, E and I**. Data are shown as the mean ± SEM.

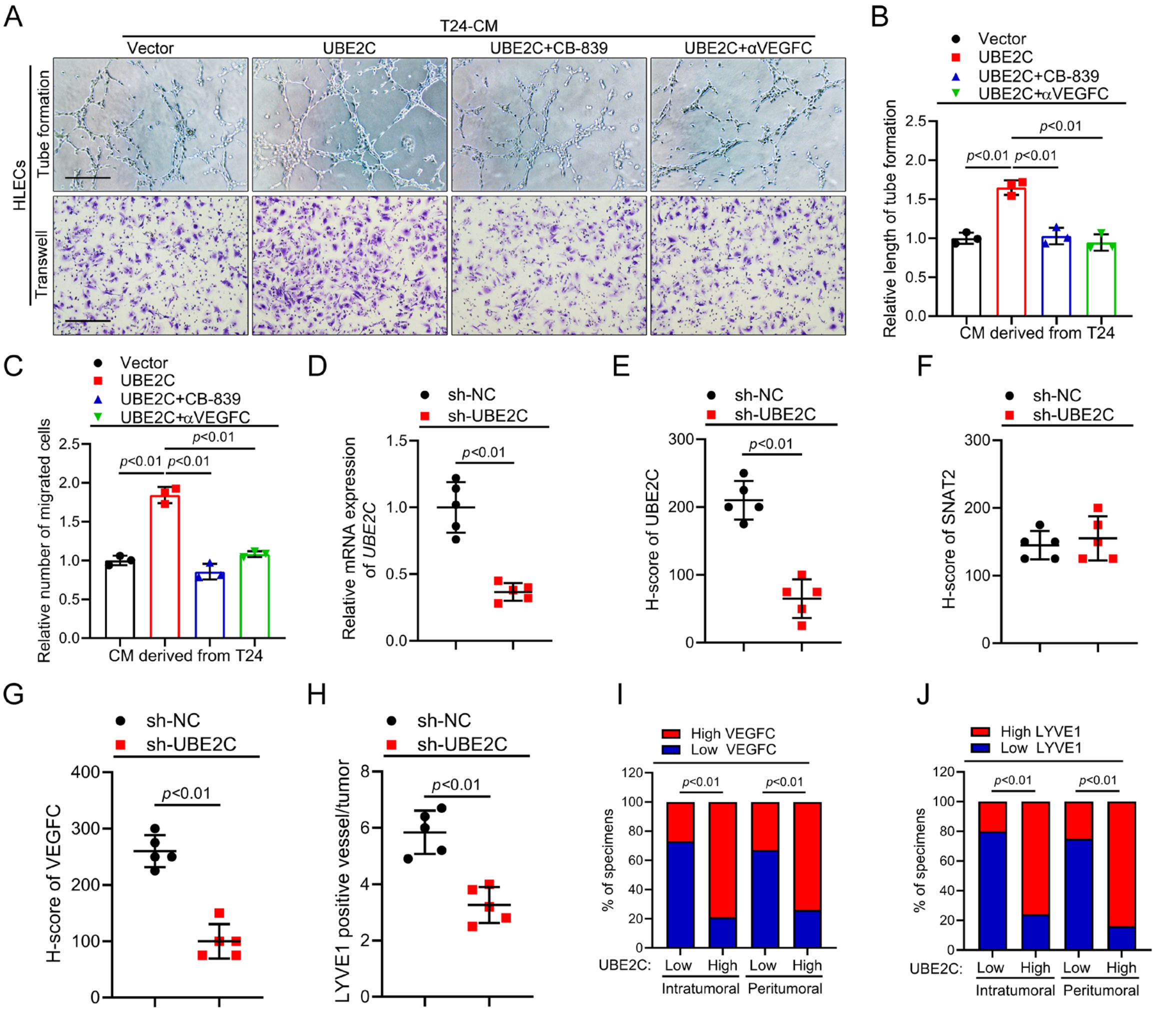

Supplemental Figure 8. UBE2C/SNAT2/VEGFC axis is crucial in BCa lymphangiogenesis and LN metastasis. (**A-C**) Representative images and quantification of tube formation and migration of HLECs treated with culture media from UBE2C-overexpressing T24 cells with or without CB-839 and αVEGFC treatment. Scale bars: 100 μm. (**D**) qRT-PCR analysis of the UBE2C expression in the tumor tissues from PDXs. (**E-H**) H-score of UBE2C, SNAT2, VEGFC and LYVE1 positive vessels from PDX tissues. (**I-J**) Quantification of UBE2C, VEGFC and LYVE1-indicated microlymphatic vessel density in both intratumoral and peritumoral regions of BCa tissues (n = 323). Significant differences

were identified through 1-way ANOVA followed by Dunnett's test in **B** and **C**; 2-tailed Student's t test in **D-H**; and the χ^2 test in **I** and **J.** Data are shown as the mean ± SEM.

Supplemental Tables

Supplemental table 1. Correlation between *UBE2C* expression and clinicopathologic characteristics of BCa patients

Characteristics	No. of cases	*UBE2C* expression		
		Low	High	*p*-value[i]
**Total cases**	323	161	162	
**Gender**				0.51
Male	281	138	143	
Female	42	23	19	
**Age**				0.66
< 65	170	87	83	
≥ 65	153	74	79	
**grade**				0.12
Low	48	29	19	
High	275	132	143	
**T stage**				0.07
0-1	118	67	51	
2-4	205	94	111	
**Lymphatic metastasis**				$<0.01^{**}$
Negative	255	139	116	
Positive	68	22	46	

Abbreviations: No. of cases = number of cases; T stage = tumor stage. [i]Chi-square Chi-square test, $^{*}p<0.05$, $^{**}p<0.01$

Supplemental table 2. Univariate and multivariate analysis of Overall Survival (OS) for *UBE2C* expression in BCa patients (n = 323)

Variables	Univariate analysis			Multivariate analysis		
	HR	95%CI	*p*-value[i]	HR	95%CI	*p*-value[i]
Age (<65 vs. ⩾65)	1.115	0.788-1.678	0.468			
Gender (Male vs. Female)	0.785	0.461-1.337	0.235			
Grade (High vs. Low)	1.445	0.808-2.587	0.215			
Lymphatic metastasis (Positive vs. Negative)	1.853	1.228-2.796	0.003**	1.630	1.051-2.527	0.029*
UBE2C expression (High vs. Low)	2.132	1.423-3.194	0.001**	2.011	1.331-3.041	0.001**

Abbreviations: HR = hazard ratio; 95% CI = 95% confidence interval; [i] Cox regression analysis, * p <0.05, ** p <0.01.

Supplemental table 3. Univariate and multivariate analysis of Disease-Free Survival (DFS) for *UBE2C* expression in BCa patients (n = 323)

Variables	Univariate analysis			Multivariate analysis		
	**HR**	**95%CI**	***p*-value[i]**	**HR**	**95%CI**	***p*-value[i]**
Age (<65 vs. ⩾65)	1.134	0.775-1.659	0.518			
Gender (Male vs. Female)	0.707	0.414-1.208	0.204			
Grade (High vs. Low)	1.597	0.876-2.912	0.127			
Lymphatic metastasis (Positive vs. Negative)	1.960	1.298-2.958	0.001^{**}	1.685	1.051-2.527	0.019^{*}
UBE2C expression (High vs. Low)	2.281	1.523-3.417	0.001^{**}	2.146	1.422-3.241	0.001^{**}

Abbreviations: HR = hazard ratio; 95% CI = 95% confidence interval; [i] Cox regression analysis, * p <0.05, ** p <0.01.

Supplemental table 4. Effect of UBE2C on popliteal LN metastasis in vivo.

**Xenograft**	**NO. metastasis LNs**	**NO. Non-metastasis LNs**	**Metastasis ratio**	***p*-value[A]**
Vector	4	8	33.33%	0.036*
UBE2C	10	2	83.33%	

[A]chi-square test, *$p<0.05$.

Supplemental table 5. Effect of CB-839 and αVEGFC on popliteal LN metastasis in vivo.

Xenograft	NO. metastasis LNs	NO. Non-metastasis LNs	Metastasis ratio	p-value^{A}
Vector	3	9	25.00%	
UBE2C	11	1	91.67%	0.002** (vs Vector)
UBE2C+CB-839	4	8	33.33%	0.009** (vs UBE2C)
UBE2C+αVEGFC	4	8	33.33%	0.009** (vs UBE2C)

^{A}chi-square test, $^{*}p<0.05$, $^{**}p<0.01$.

Supplemental table 6. Primer and small interfering RNAs (siRNAs) used in the experiments.

Gene	Sequence (5’-3’)	Application
UBE2C	F: AGTGGCTACCCTTACAATGCG	qRT-PCR
	R: TTACCCTGGGTGTCCACGTT	
GAPDH	F: GGAGCGAGATCCCTCCAAAAT	qRT-PCR
	R: GGCTGTTGTCATACTTCTCATGG	
si-UBE2C#1	Sense: GACCUGAGGUAUAAGCUCUTT	siRNA
	Antisense: AGAGCUUAUACCUCAGGUCTT	
si-UBE2C#2	Sense: GUAUGAUGUCAGGACCAUUTT	siRNA
	Antisense: AAUGGUCCUGACAUCAUACTT	
si-NEDD4L#1	Sense: CAUGUUCUGCAUAGACGAA	siRNA
	Antisense: UUCGUCUAUGCAGAACAUG	
si-NEDD4L#2	Sense: GAAGAGUCCUAUCGGAGAA	siRNA
	Antisense: UUCUCCGAUAGGACUCUUC	

Supplemental table 7. Antibody used in the experiments.

Product	Source	No. of Catalogue
**Primary antibody:**		
**Western blot:**		
Anti-UBE2C	Cell Signaling Technology	14234<br>RRID: AB_2722751
Anti-GAPDH	Abcam	ab8245<br>RRID: AB_2107448
Anti-FLAG	Abcam	ab205606<br>RRID: AB_2916341
Anti-HIS	Abcam	ab18184<br>RRID: AB_444306
Anti-HA	Abcam	ab9110<br>RRID: AB_307019
Anti-K63 Poly-Ub	Abcam	ab179434<br>RRID: AB_2895239
Anti-NEDD4L	Immunoway	YN3050<br>RRID: AB_3076204
Anti-SNAT2	Immunoway	YT4354<br>RRID: AB_3076205
Anti-Na/K ATPase	Abcam	ab76020<br>RRID: AB_1310695
Anti-LAMIN B1	Abcam	ab16048<br>RRID: AB_443298
Anti-EPSIN1	ABclonal	A20872<br>RRID: AB_3076206
Anti-VEGFC	Abcam	Ab9546<br>RRID: AB_2241408
**Immunofluorescence:**		
Anti-UBE2C	Abcam	ab252940<br>RRID: AB_2910263
Anti-LYVE1	Abcam	ab219556<br>RRID: AB_2884014
Anti-SNAT2	Immunoway	YT4354<br>RRID: AB_3076205
Anti-RAB5	Abcam	ab218624<br>RRID: AB_2892717
Anti-Pan-CK	Abcam	ab86734<br>RRID: AB_10674321
**IHC:**		
Anti-UBE2C	Abcam	ab252940<br>RRID: AB_2910263
Anti-LYVE1	Abcam	ab219556<br>RRID: AB_2884014
Anti-VEGFC	Abcam	Ab9546<br>RRID: AB_2241408

Anti-SNAT2	Immunoway	YT4354<br>RRID: AB_3076205
**IP:**		
Anti-FLAG	Abcam	ab205606<br>RRID: AB_2916341
Anti-HIS	Abcam	ab18184<br>RRID: AB_444306
Anti-SNAT2	Immunoway	YT4354<br>RRID: AB_3076205
**ELISA:**		
VEGFC	Abcam	ab9546<br>RRID: AB_2241408
**Secondary antibody:**		
**Western blot:**		
Anti-rabbit IgG-HRP	Cell Signaling Technology	7074<br>RRID: AB_2099233
Anti-mouse IgG-HRP	Cell Signaling Technology	7076<br>RRID: AB_330924
**Immunofluorescence:**		
Alexa Fluor 594	Abcam	Ab150080<br>RRID: AB_2650602
Alexa Fluor 488	Abcam	Ab150077<br>RRID: AB_2630356

Supplemental Methods

Protein extraction

BCa cells were harvested and washed 3 times with PBS, followed by complete lysis with RIPA lysis buffer (Thermo Fisher Scientific, USA, Cat#89901) supplemented with 1% protease inhibitor (Thermo Fisher Scientific, Cat#87786) and 1% phosphatase inhibitor (Thermo Fisher Scientific, Cat#78427) at 4°C for 30 min. The lysate was centrifuged at 12,000g for 30 minutes. For cellular fractions protein extraction, cell membrane and endosomal proteins were extracted using Plasma Membrane Protein Isolation Kit (Invent, USA, Cat#SM-005) and Endosome Isolation Kit (Invent, Cat#ED-028), respectively. A BCA protein assay kit (Thermo Fisher Scientific, Cat#A55864) was used to measure the protein concentration.

Western blotting

The expression of the indicated proteins was detected by western blotting analysis. Total proteins were separated by 10% SDS-PAGE and then transferred to a polyvinylidene fluoride membrane (Millipore Sigma, USA, Cat#3010040001). After blocking with 5% BSA for 1 h, the membrane was incubated with the indicated primary antibodies overnight at 4°C, followed by a 1 h incubation with HRP-conjugated secondary antibodies at room temperature. Immunoblots of the target proteins were detected using an enhanced chemiluminescence (ECL) kit (Thermo Fisher Scientific, Cat#32209) and quantified with ImageJ software (ImageJ, RRID:SCR_003070) (NIH, Bethesda, MD, USA). The detailed antibodies used in the experiments are listed in Supplemental Table 7, and the full uncut original images are

shown in Full unedited blot.

IHC

For IHC analysis, the paraffin-embedded tissue sections were heated in an incubator for 2 hours at 65°C before being dewaxed with dimethylbenzene and hydrated with gradient alcohols. Subsequently, the sections were placed in heated EDTA buffer to retrieve the antigen and incubated with peroxidase inhibitors to block endogenous peroxidase activity. After blocking with normal goat serum for 30 min, the sections were incubated with the indicated primary antibodies overnight at 4°C and with secondary antibodies for 30 min at room temperature. Finally, the sections were stained with 3,3'-diaminobenzidine (DAB) and hematoxylin, and images were acquired with a Nikon eclipse 80i (Nikon, Tokyo, Japan) and analyzed with ImageJ software (RRID:SCR_003070). Supplemental Table 7 lists the antibodies used in the experiments.

Histology evaluation of tissue sections

Regarding the examination of UBE2C expression, the percentage of tumor cells that stained positively was classified into the following categories: 0 (no positive staining), 1 (0-10 percent positive), 2 (10-30 percent positive), 3 (30-70 percent positive), and 4 (over 70 percent positive). A four-point rating system was used to indicate the degree of staining: 1 represented no staining, 2 mild staining, 3 moderate staining, and 4 strong staining. A possible score of 0, 1, 2, 3, 4, 6, 8, 9, 12, and 16 could be obtained by multiplying the positive percentage by the staining intensity to calculate the Staining Index (SI). The median value, specifically SI = 8, was then determined to be the threshold. Samples exhibiting low

expression were classified as having a SI < 8, while samples exhibiting high expression were classified as having a SI ≥ 8. About the quantification of LYVE1, Image J software (NIH) determined how many vessels had positive staining in three random fields for each section. The cut off value was used to define the median value.

qRT-PCR

According to the manufacturer's instructions, the total RNA was extracted from the cells by using Total RNA Extraction Reagent (EZBioscience, USA, Cat#EZB-TZ1). Subsequently, the RNA samples were reverse transcribed with the Hiscript III Reverse Transcriptase Kit (Vazyme, Nanjing, China, Cat#R312-01). The expression of the indicated genes was measured by qRT-PCR with the ChamQ™ Universal SYBR qPCR Master Mix Kit (Vazyme, Cat#Q711-02). The detailed sequences of the primers used are listed in Supplemental Table 6.

Transwell assays

Transwell assays were used to evaluate the invasive and migratory ability of BCa cells and the migratory ability of HLECs. To perform migration assays, the lower chamber was filled with 700 μl of medium containing 10% FBS, while the upper chamber (Corning Costar Corp, USA, Cat#3422) was filled with 300 μl of suspension containing either 1×10^5 BCa cells or 3×10^4 HLECs. To perform invasion assays, the membranes of the upper chambers were first coated with Matrigel (BD Biosciences, USA, Cat#356234). The indicated cells were subsequently plated following the same procedures as those used for the migration assays. The migrated cells were fixed and stained with 0.1% crystal violet after they had incubated for 4 hours for HLECs, 6 hours for T24 cells, or 12 hours for UM-UC-3 cells at 37°C and 5%

CO_2. Images were captured with a Nikon eclipse 80i (Nikon, Japan), and the number of migrated cells in five random fields was counted with ImageJ software (RRID:SCR_003070).

Tube formation assays

Tube formation assays were performed to evaluate the tube formation ability of HLECs. Briefly, 24-well plates were filled with a 400 µl mixture containing Matrigel (BD Biosciences, Cat#356234) and FBS-free ECM at a 1:2 ratio. The plates were then incubated overnight at 37°C. Subsequently, the Matrigel-covered wells were seeded with 300 µl of suspension containing 1×10^5 of the indicated HLECs and incubated for 4 hours. Finally, images of the formed lymphatic vessels were obtained via inverted fluorescence microscopy (Olympus IX73, Japan), and the tube length was measured using ImageJ (RRID:SCR_003070).

Immunofluorescence

For cell immunofluorescence, cells were fixed with 4% paraformaldehyde for 15 min and permeabilized with 0.5% Triton X-100 (Sigma-Aldrich, USA, Cat#9036-19-5) for 15 min. After the cells were blocked for 1 h at 37°C with normal goat serum, they were incubated with the indicated primary antibody at 4°C overnight. The cells were incubated with the corresponding fluorescent secondary antibodies for 30 min at room temperature, and the nuclei were stained with DAPI for 15 min. Finally, the cells were imaged using laser scanning confocal microscopy (LSM710, Zeiss, Pleasanton, CA, USA). Supplemental Table 7 lists the antibodies used in the experiments.

Flow cytometry

A total of 5×10^5 cells were harvested, washed 3 times with PBS and incubated with the

indicated fluorescent antibodies for 30 minutes at room temperature. After washing with PBS, the samples were resuspended in 200 μl of PBS and analyzed by flow cytometry. The cells were initially gated based on side scatter and forward scatter parameters to exclude debris. Subsequently, the gating was refined using the blank control group to exclude negative cells. Supplemental Table 7 lists the antibodies used in the experiments.

Protein half-life measurement

Cells were treated with CHX (Selleck, Guangzhou, China, Cat#S7418) at a concentration of 50 μg/ml and collected at 0, 0.5, 1, 2, and 4 h. Time course protein levels of SNAT2 were assessed using western blotting.

ATP assay

ATP production was measured with an ATP assay kit (Beyotime, Guangzhou, China, Cat#S0026B) following the manufacturer's instructions. cells were lysed and centrifuged. Supernatants and standard substances were mixed with the ATP detection working solution in a 96-well plate. All values were normalized to the protein concentration.

Cell viability

To determine the cell viability of different cell group, we seeded cells into 96-well at 3000 cells/well. Cell viability of each group was determined using a Cell Counting Kit-8 (CCK-8) (MedChemExpress, USA, Cat#HY-K0301) according to the manufacturer's instructions.

ELISA

For the quantitation of secreted VEGFC, ELISAs were conducted with the Human VEGFC ELISA Kit (Abcam, England, Cat#ab100664) according to the manufacturer's instructions.

Briefly, a 96-well plate was filled with 100 μL of each standard or sample and incubated for 2.5 hours at room temperature. After washing with wash solution, each well, except for the blank wells, was filled with 100 μL of biotinylated VEGFC detection antibody and incubated for 1 hour at 37°C. This was followed by a 45-minute incubation with HRP-streptavidin solution. Finally, the reaction was stopped by adding 50 μl of stop solution, and the OD was measured at 450 nm with a SYNERGY H1 microplate handler (Bio-Tek, USA).

High-throughput sequencing

Total RNA was extracted with TRIzol Reagent (Invitrogen, USA, Cat# 15596026). The mRNA libraries were established and sequenced on a HiSeq 4000 platform by Gene Denovo Biotechnology Co., Ltd. (Guangzhou, China).

PLA

A PLA was used to detect protein interactions in BCa cells. Briefly, BCa cells were seeded in a confocal dish and fixed with 4% paraformaldehyde for 15 minutes. Subsequently, the PLA was performed according to the instructions of the DUOLINK®: PLA Kit (Sigma-Aldrich, Cat#DUO92202) with the indicated antibodies. Images were captured using a laser scanning confocal microscope (Zeiss, USA).

Silver staining

The protein specimens were obtained through coimmunoprecipitation assays, and comparable volumes of these specimens were subjected to electrophoretic separation using a 10% SDS-PAGE gel. Subsequently, the separated proteins underwent a washing process and were subjected to silver staining employing a Silver Stain kit (Thermo Scientific, Cat#24600),

following the manufacturer's instructions.
