# Supplementary material for: UBE2C-induced crosstalk between mono- and polyubiquitination of SNAT2 promotes lymphatic metastasis in bladder cancer
Source: J Clin Invest. 2024 Jul 1;134(13):e179122. doi: 10.1172/JCI179122 (PMC11213464; doi:10.1172/JCI179122)

Fig1C

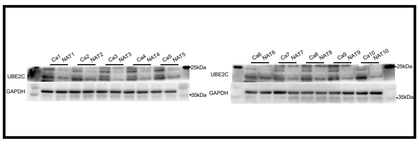

Fig2A Fig2B

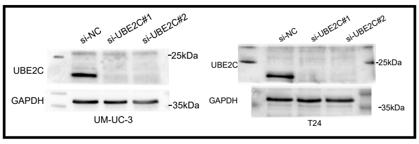

Fig2C Fig2D

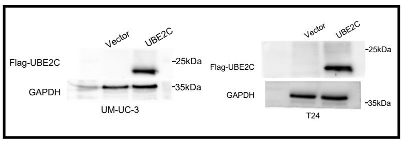

Fig3B

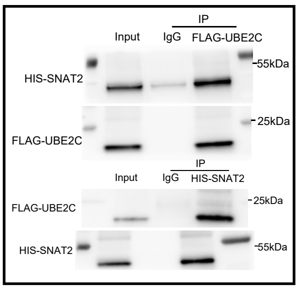

Fig3D

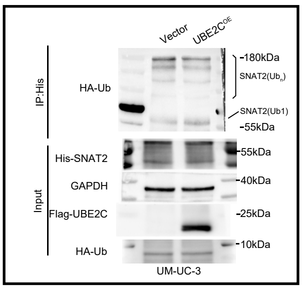

Fig3E

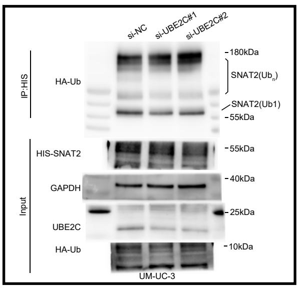

Fig3F

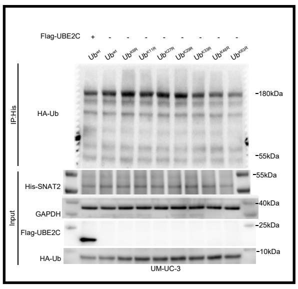

Fig3G

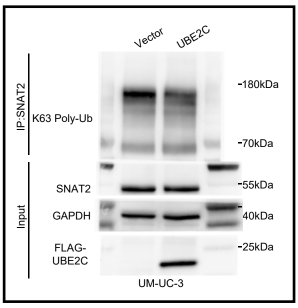

Fig3H

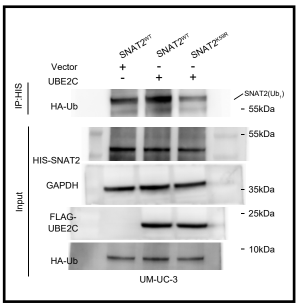

Fig3I

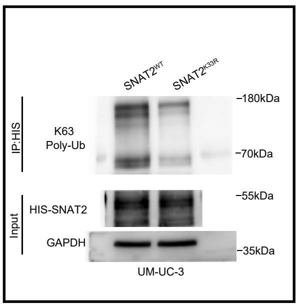

Fig3J

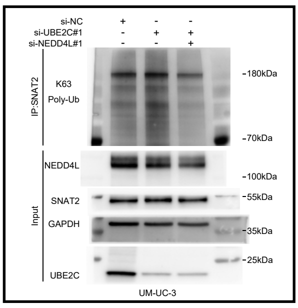

Fig4C

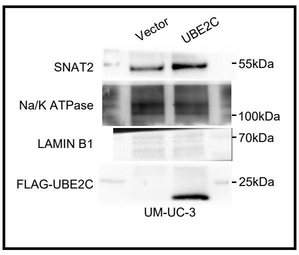

Fig4E

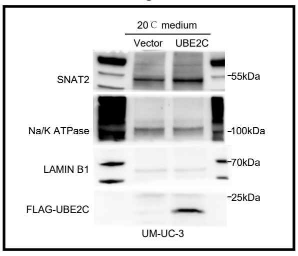

Fig4F

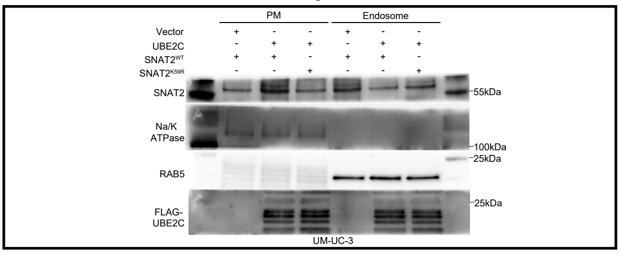

Fig4I

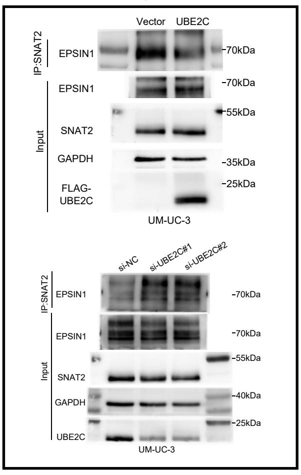

Fig4J

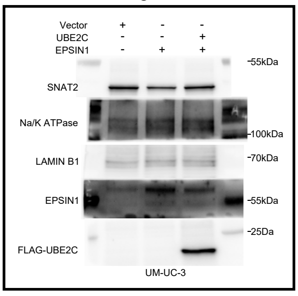

Fig4M

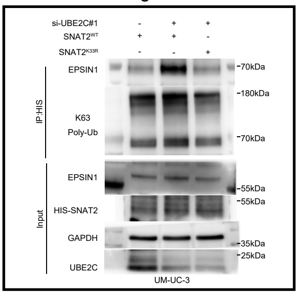

Fig4N

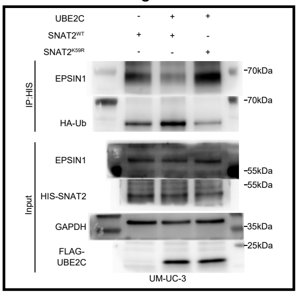

FigS5F

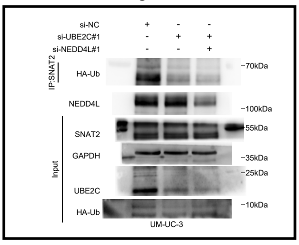

FigS6 A and B

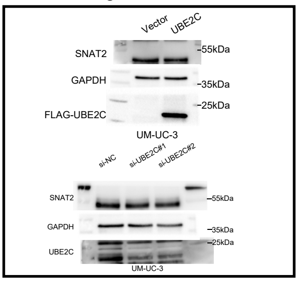

FigS6N

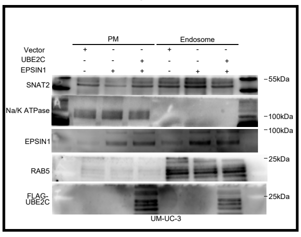

FigS2A

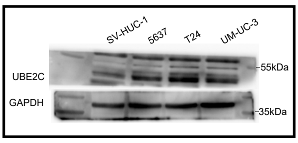

FigS5G

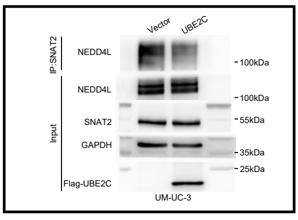

FigS6C

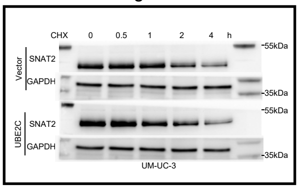

FigS7J

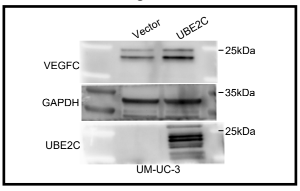

FigS4F

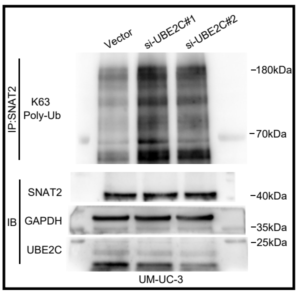

FigS7L

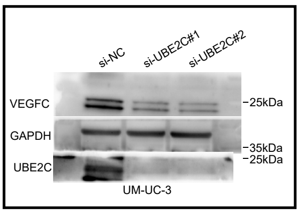

FigS5H

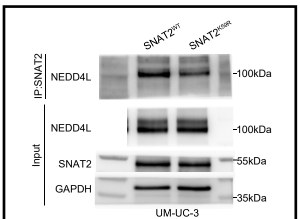

FigS6H

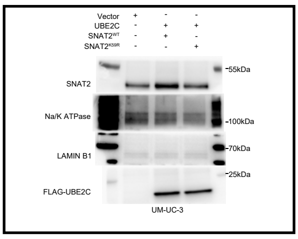

FigS5D

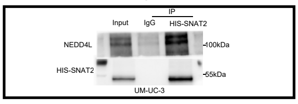

FigS5H

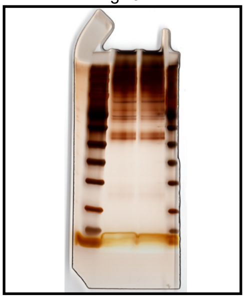

FigS7N

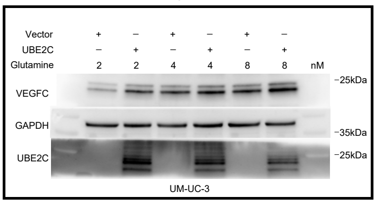

FigS7P

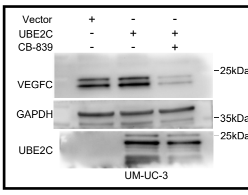

Fig2L

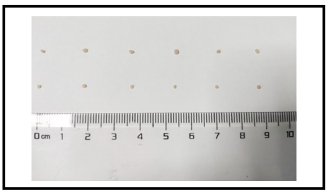

Supplement: Unedited blot and gel images [file jci-134-179122-s218.pdf]
